# The VE-cadherin/AmotL2 mechanosensory pathway suppresses aortic inflammation and the formation of abdominal aortic aneurysms

**DOI:** 10.1038/s44161-023-00298-8

**Published:** 2023-06-29

**Authors:** Yuanyuan Zhang, Yumeng Zhang, Evelyn Hutterer, Sara Hultin, Otto Bergman, Solrun Kolbeinsdottir, Hong Jin, Maria J. Forteza, Daniel F. J. Ketelhuth, Joy Roy, Ulf Hedin, Martin Enge, Ljubica Matic, Per Eriksson, Lars Holmgren

**Affiliations:** 1 Department of Oncology-Pathology, BioClinicum, Karolinska Institutet, Stockholm, Sweden; 2 Department of Medicine Solna, BioClinicum, Karolinska Institutet, Karolinska University Hospital, Stockholm, Sweden; 3 Department of Molecular Medicine and Surgery, Karolinska Institutet, Karolinska University Hospital, Stockholm, Sweden; 4 Department of Cardiovascular and Renal Research, Institutet of Molecular Medicine, University of Southern Denmark, Odense, Denmark

**Keywords:** Cadherins, Inflammation, Aneurysm

## Abstract

Endothelial cells respond to mechanical forces exerted by blood flow. Endothelial cell–cell junctions and the sites of endothelial adhesion to the matrix sense and transmit mechanical forces to the cellular cytoskeleton. Here we show that the scaffold protein AmotL2 connects junctional VE-cadherin and actin filaments to the nuclear lamina. AmotL2 is essential for the formation of radial actin filaments and the alignment of endothelial cells, and, in its absence, nuclear integrity and positioning are altered. Molecular analysis demonstrated that VE-cadherin binds to AmotL2 and actin, resulting in a cascade that transmits extracellular mechanical signals to the nuclear membrane. Furthermore, the endothelial deficit of AmotL2 in mice fed normal diet provoked a pro-inflammatory response and abdominal aortic aneurysms (AAAs). Transcriptome analysis of human AAA samples revealed a negative correlation between AmotL2 and inflammation of the aortic intima. These findings offer insight into the link between junctional mechanotransduction and vascular disease.

## Abstract (web summary)

Zhang et al. identified a protein complex that includes AmotL2 and forms a connection between VE-cadherin and actin filaments to the nuclear lamina in endothelial cells, thereby impacting cell alignment and nuclear integrity. The deletion of AmotL2 was found to provoke inflammation and abdominal aortic aneurysms, suggesting a linkage between junctional mechanotransduction and vascular disease.

## Main

The blood vessel wall is lined with a thin layer of vascular endothelial cells (ECs), which form a barrier between the blood and tissues. These cells differ in biochemical characteristics depending on their localization in arteries or veins as well as on the organ in which they reside. The endothelium is continuously exposed to the shear stress exerted by blood flow. Understanding how ECs respond to shear stress is of importance as it has implications for the development of vascular diseases. Indeed, since the 1870s, it has been postulated that disturbed blood flow exerted on the vessel wall may be a trigger of atherosclerosis. Also, low wall shear stress has been associated with abdominal aortic aneurysm (AAA) rupture^1^. AAA is characterized by localized medial and adventitial inflammation and dilatation of the abdominal aorta and is prevalent in men over 65 years of age, with high morbidity and mortality^2^. By contrast, areas of laminar flow appeared relatively protected against the development of the inflammatory disease^3^.

To explore how the mechanotransductive pathways mediate protection or activation of the vascular disease is of clear importance^4–16^. To date, several mechanosensory pathways have been identified that relay external mechanical forces to the endothelial lining^17^. In vitro, flow-induced endothelial alignment is dependent on the activation of GTPases and consequent actin reorganization. In vivo, it has been shown that endothelial junctional protein complexes, including PECAM1(Cd31), VE-cadherin and VEGFR2, play an important role in the adaptive response to shear stress^16^. However, it is still not clear how mechanical forces exerted by the blood flow are transmitted from the junctions via the cytoskeleton into the cell.

Studies of the angiomotin (Amot) protein family may provide important insights into this aspect. This is a family of scaffold proteins that link membrane receptors to the actin cytoskeleton and polarity proteins and are implicated in modulating the Hippo pathway^18–24^. We recently showed that one of its members, AmotL2 (p100 isoform), is associated with the VE-cadherin complex in ECs and E-cadherin in epithelial cells^23,25^. Silencing of AmotL2 in zebrafish, mouse or cells in vitro results in a loss of radial actin filaments that run perpendicular to the outer cell membrane. These actin filaments mechanically connect cells via binding to junctional cadherins and, thereby, transmit force. Conditional silencing of AmotL2 in the endothelial lineage of mice inhibits expansion of the aorta during the onset of circulation, resulting in death in utero at embryonic day 10 (ref. ^23^). In this study, we analysed the role of AmotL2 in controlling junctional and cytoskeletal components during the alignment of arterial ECs exposed to laminar flow. Here we present a mechanical transduction pathway active in arteries that is protective against vascular inflammation as well as the formation of AAAs.

## Results

### AmotL2 is essential for arterial endothelial alignment

**Fig. 1:**
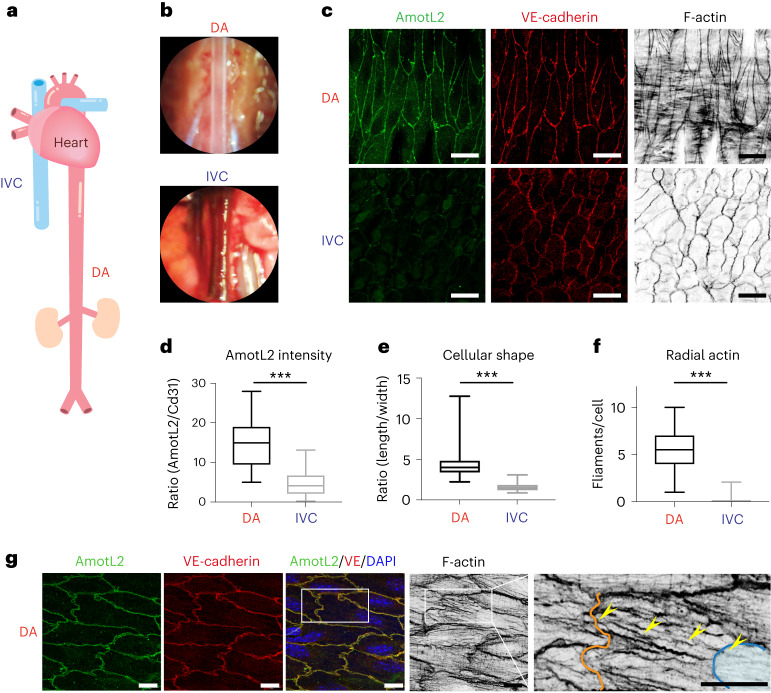
AmotL2 is primarily expressed in aortic ECs. **a**, Schematic of major blood vessels connected to the heart. **b**, Anatomical images of the DA (taken after PBS perfusion) and IVC under microscopy during the dissection process. **c**, Representative images of DA and IVC from adult mice aged 7–9 months (*n* = 3 from three independent tissue harvests) used for whole-mount staining. Both vessels were stained with immunoaffinity purified AmotL2 antibodies (green), VE-cadherin (red) and phalloidin (in grayscale). **d**, Box plot showing quantification of the fluorescence intensity of AmotL2 in co-localization with Cd31 in *amotl2*^*ec+/ec+*^ DA (*n* = 3) using ImageJ. **e**, Box plot depicting the difference in cell length/width ratio in *amotl2*^*ec+/ec+*^ DA (166 cells were quantified) and IVC (149 cells). **f**, Quantification of macroscopic radial actin filaments per EC in DA and IVC. More than three mice and at least four images per mouse were analysed for each group. **g**, High-resolution images of DA stained with AmotL2 (green), VE-cadherin (red) and phalloidin (in grayscale). The blue line outlines the nucleus, and the orange line indicates the cellular membrane. Yellow arrows follow the actin filaments that cross from the cellular membrane to the nucleus. Three mice were examined for this purpose. Scale bars, 20 µm (**c**) and 10 µm (**g**). ^***^P < 0.001. Source data

**Extended Data Fig. 1:**
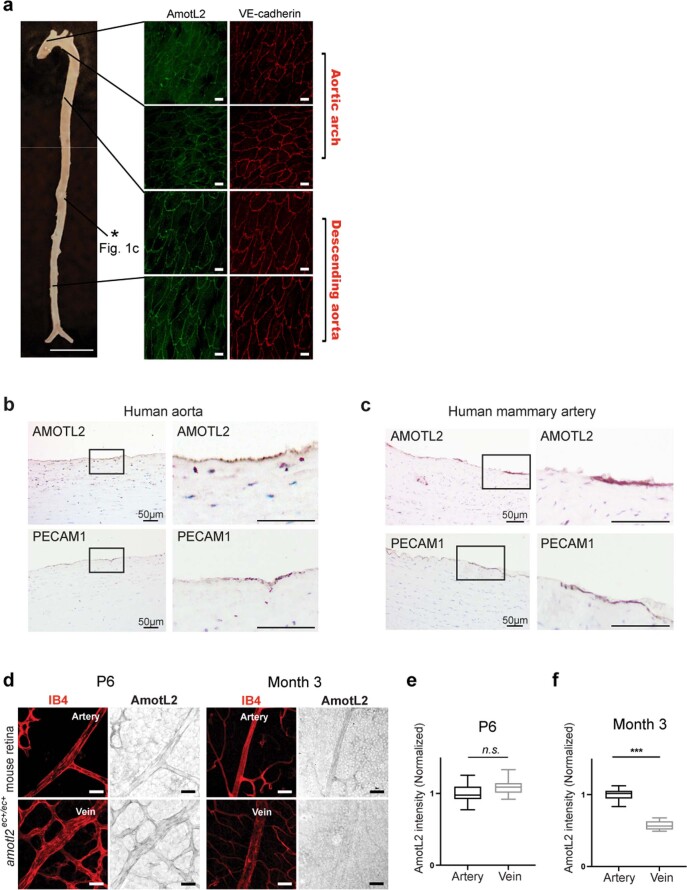
AmotL2 expression pattern in mouse aorta and retina. **a**, Representative images of whole aorta with AmotL2 (green) and VE-cadherin (red) staining. The mice were aged 7–9 months. Scale bars: 1000 µm (schematic) and 10 µm (IF images). **b,c**, IHC staining on the paraffin sections of human aorta (**b**) and mammary artery (**c**) from healthy donors (n = 2 for each type of tissue) stained with PECAM1 and AMOTL2, respectively. Framed area is magnified on the right. Scale bars: 50 µm. **d**, *amotl2*^*ec+/ec+*^ retinas were stained with IB4 (red) and AmotL2 (grey) at postnatal day 6 and month 3. Representative images of arteries and veins are presented. Scale bars: 25 µm (left panel) and 50 µm (right panel). The quantification of Amotl2/IB4 intensity in arteries and veins (normalized to the average of that in arteries) at postnatal day 6 (**e**) and month 3 (**f**) is shown in box plots. The retinas from three mice in each age group were stained and at least two images per retina were analysed. *n.s*., not significant. ^***^P < 0.001. Source data

We analysed the expression pattern of AmotL2 in mouse descending aorta (DA, both thoracic and abdominal parts) and the inferior vena cava (IVC) as shown in Fig. 1a,b. ECs of the DA were typically elongated and aligned in the direction of blood circulation and contained radial actin filaments that were connected to the cellular junctions (Fig. 1c,g and Extended Data Fig. 1a). AmotL2 localized to aortic EC junctions as previously reported^23^. In contrast, ECs of the IVC exhibited a more rounded cellular shape with no or few detectable radial actin filaments as well as lower expression levels of AmotL2 (Fig. 1c). Box plots in Fig. 1d–f represent the statistically significant difference between the DA and IVC with regard to AmotL2 expression, cellular shape and the presence of radial actin filaments. Interestingly, radial actin filaments in DA were visualized across adjacent ECs and overlapped with the nuclei as visualized by high-resolution microscopy (Fig. 1g). Immunohistochemical staining of human aorta and mammary artery cross-sections showed specific expression of AmotL2 in ECs rather than smooth muscle cells (Extended Data Fig. 1b,c).

In addition, we mapped AmotL2 expression in retinal vasculature of neonatal and adult mice (Extended Data Fig. 1d). AmotL2 was expressed at similar levels in arteries and veins at postnatal day 6, whereas, in adult mice (3 months), AmotL2 was primarily expressed in retinal arteries (Extended Data Fig. 1e,f).

**Fig. 2:**
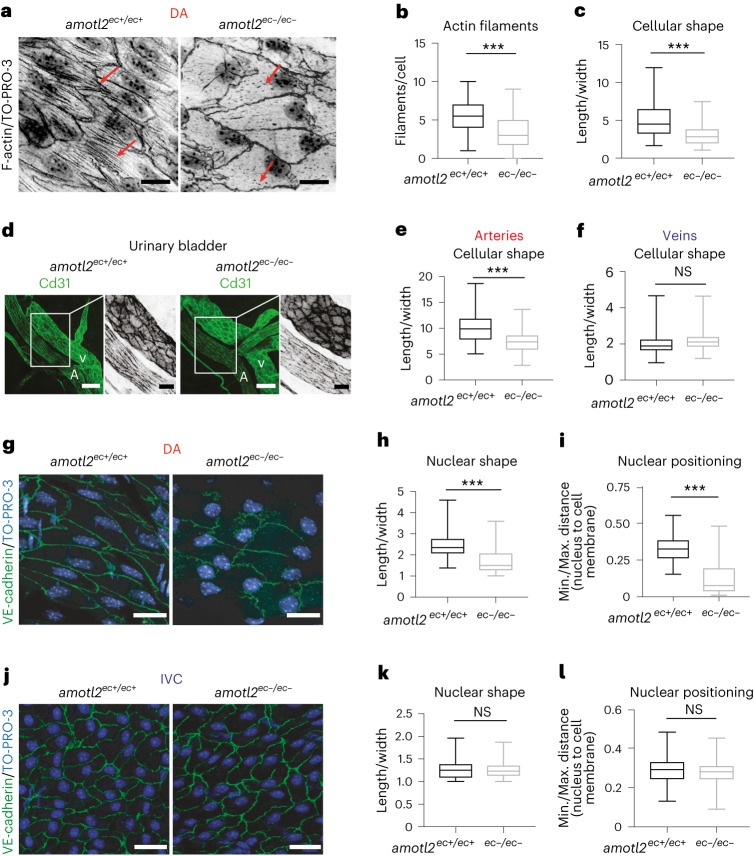
AmotL2 is required for cell alignment and nuclear positioning. **a**, Whole-mount IF staining of *amotl2*^*ec+/ec+*^ and *amotl2*^*ec*−*/ec*−^ DA (7–9-month-old mice) with phalloidin and TO-PRO-3 showing F-actin and nuclei (in grayscale), respectively. The red arrow points to the actin filaments. The staining was performed in three independent experiments. **b**,**c**, Quantification of cell length/width ratio (**b**, 154 cells) and macroscopic radial actin filaments per cell (**c**, 179 cells) in aortic ECs (six mice enrolled in each group). **d**, IF staining of bladder vasculature in *amotl2*^*ec+/ec+*^ and *amotl2*^*ec*−*/ec*−^ mice with Cd31 indicating ECs (green). Six mice in each group were analysed for **d**–**f**. **e,f**, Quantifications of EC shape in bladder arteries (**e**) and veins (**f**). The boxed area in the left panel is magnified in the middle and right panels. The white character V and A are abbreviations for vein and artery, respectively. **g**, Whole-mount IF staining of *amotl2*^*ec+/ec+*^ and *amotl2*^*ec*−*/ec*−^ DAs with VE-cadherin (green) and TO-PRO-3 showing nuclei (blue). The staining was performed three times independently. **h**, Quantification of nuclear length/width ratio in aortic ECs (*n* ≥ 6 in each group; *amotl2*^*ec+/ec+*^ 146 cells and *amotl2*^*ec/ec*−^ 101 cells). Along with the long axis of the EC, the closest distance between the nuclei and the one end of the cell was measured and normalized to the full cell length. The ratio is depicted in box plot **i** (*amotl2*^*ec+/ec+*^ 86 cells and *amotl2*^*ec/ec*−^ 142 cells). **j**, Representative images on IVC of *amotl2*^*ec+/ec+*^ and *amotl2*^*ec*−*/ec*−^ mice with VE-cadherin (green) and TO-PRO-3 (blue). Staining was examined in three independent experiments. Quantification of the nuclear shape and nuclear positioning of ECs in IVC are presented in **k** and **l** (*n* = 5 from three independent tissue harvests). Scale bars, 10 µm (**a**,**g**,**j**) and 20 µm (**d**). ^***^P < 0.001. Source data

**Extended Data Fig. 2:**
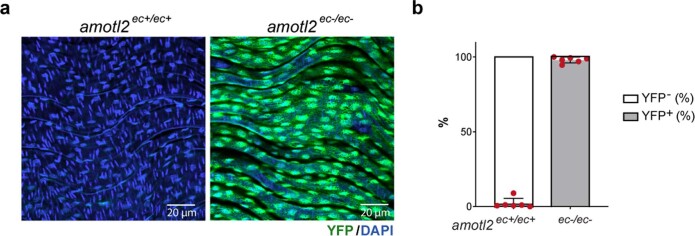
Recombination efficiency in *amotl2*^*flox/flox*^ Cdh5(PAC)^CreERT2^ ROSA26-EYFP mice. **a**,**b**, GFP immunofluorescence staining (green) used for visualizing the YFP reporter, as a marker for Cre-recombinase expression of DAs in *amotl2*^*ec+/ec+*^ and *amotl2*^*ec*−*/ec*−^ Cdh5(PAC)^CreERT2^ ROSA26-EYFP mice. The mice were aged of 7–9 months. The staining has been performed in six mice in each group from three independent experiments. %YFP^+^/YFP^−^ quantification of *amotl2*^ec+/ec+^ (1031 cells) and *amotl2*^*ec*−*/ec*−^ (1044 cells) DAs was presented in **b**. Data are presented as mean ± s.d. Scale bars: 20 µm. Source data

We previously showed that AmotL2 is required for the formation of radial actin filaments both in epithelial cells and endothelial cells^23,25^. The term radial actin refers to filaments that connect to adherence junctions and are formed perpendicular to the cell membrane^26^. The preferential expression of AmotL2 in ECs of the aorta raised the possibility that AmotL2 controlled arterial EC shape via the formation of radial actin fibres. To address this question, we used a genetic deletion approach to silence *amotl2* gene expression specifically in the endothelial lineage, as previously reported^27^. In this model system, *amotl2*^flox/flox^ mice were crossed with Cdh5(PAC)^CreERT2^ transgenics as well as ROSA26-EYFP reporter mice^23^, hereafter referred to as *amotl2*^*ec/ec*^. This crossing enables efficient inducible conditional recombinase expression and subsequent *amotl2* knockout (KO) in ECs (*amotl2*^*ec−/ec−*^) after tamoxifen injections as well as quantification of recombination efficiency by YFP expression (Extended Data Fig. 2a,b). Adult mice (7–9 months old) were euthanized 1 month after tamoxifen injections, and aortas were dissected and analysed by whole-mount immunostaining. Inactivation of AmotL2 in the DA resulted in the loss of radial actin filaments and altered cell shape (Fig. 2a, quantification in Fig. 2b,c). This observed phenotype change appeared to be arterial-specific as similar effects were observed in arterial but not venous ECs of the urinary bladder (Fig. 2d, quantification in Fig. 2e,f).

The nucleus is the largest organelle of the EC. As such, it is exposed to the hemodynamic drag by the blood flow. In response to shear stress, EC as well as EC nuclei elongate, and nuclei orient themselves relative to the direction of flow. Nuclear positioning as well as alignment was previously shown to be dependent on the association to microfilaments as well as the tubulin network^28^. In *amotl2*^ec+/ec+^ mice, nuclei of ECs of the DA were elongated and orientated in parallel with cell alignment in the direction of blood flow. However, in AmotL2-deficient ECs, the nuclei were more rounded with irregular shapes and positioned close to the cell–cell junctions downstream of the flow direction (Fig. 2g, quantification in Fig. 2h,i). These changes in nuclear shape and positioning were not observed in the IVC (Fig. 2j, quantification in Fig. 2k,l). Taken together, these data show that AmotL2 is required for EC elongation as well as positioning of the EC nucleus.

### AmotL2 expression is required for arterial response to flow

**Fig. 3:**
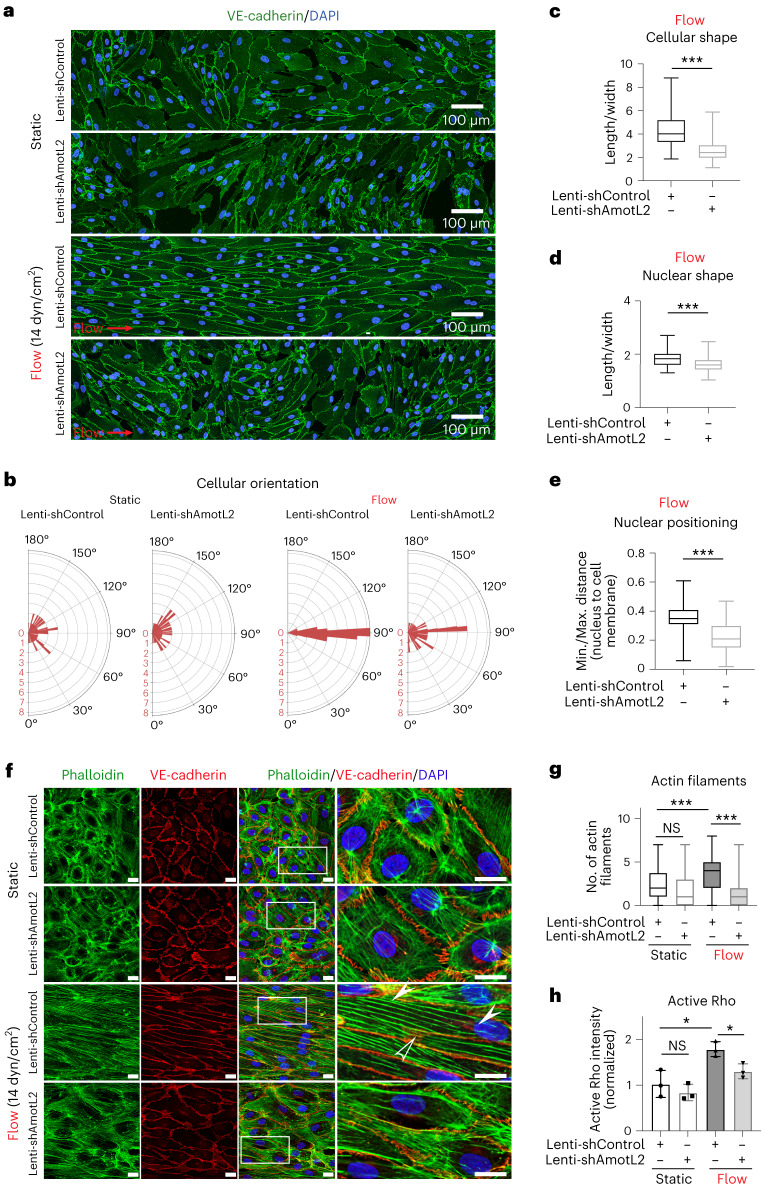
AmotL2 is essential for flow-induced cell alignment in vitro. **a**, IF staining of VE-cadherin (green) and Hoechst (blue) of HAoECs treated by scrambled or AmotL2 lenti-shRNA under static or flow (14 dynes per cm^2^ (dyn/cm^2^), 48 h) conditions. The flow direction is indicated by the red arrow. Similar results were observed in five independent experiments. Quantification analysis of cellular orientation is shown in polar bar charts in **b**. Cell length/width ratio (**c**, shControl *n* = 137, shAmotL2 *n* = 104), nuclear length/width ratio (**d**, shControl *n* = 50, shAmotL2 *n* = 50) and nuclear positioning (**e**, shControl *n* = 71, shAmotL2 *n* = 55) under flow condition were quantified and are presented in box plots. **f**, High-resolution IF staining with VE-cadherin (red), phalloidin (green) and Hoechst (blue) on HAoECs. The hollow arrow indicates the actin filament crossing neighbouring cells, and the solid arrows follow the long actin filament overlapping with the nucleus. Scale bars, 20 µm. The number of radial actin filaments crossing the nuclei (*n* = 100 cells in each group from five independent experiments) is shown in the box plot (**g**). **h**, Active Rho/all Rho input in HAoECs as quantified in different groups after normalization to the level of the static control. Data were gathered from three independent experiments and are presented as mean ± s.d. ^***^P < 0.001. Source data

**Extended Data Fig. 3:**
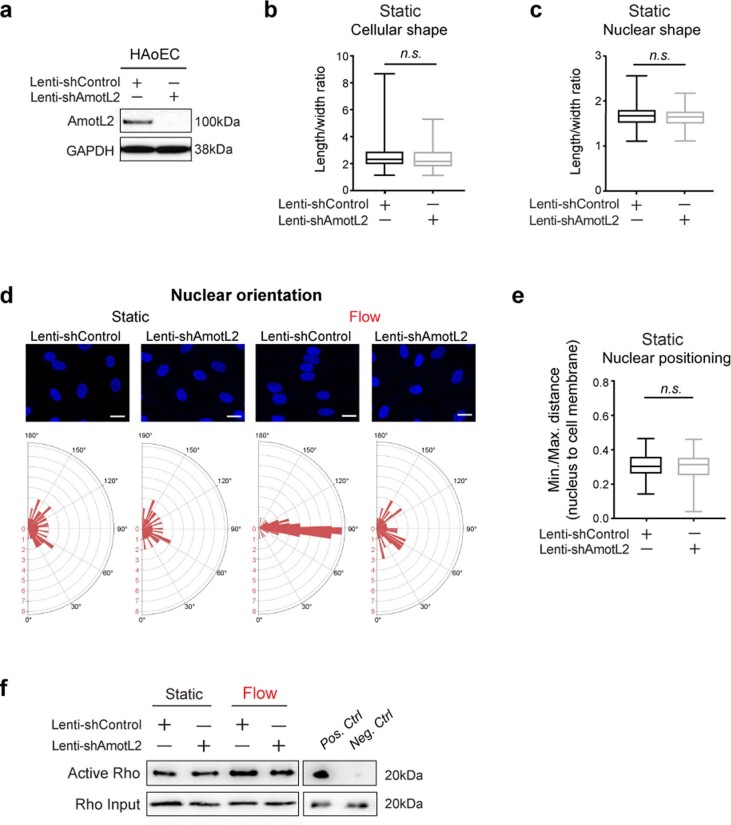
Effect of AmotL2 knock-down on HAoEC *in vitro*. **a**, WB analysis of whole cell lysates of confluent HAoECs transfected by scrambled control or AmotL2 lenti-shRNA. Quantification of cellular length/width ratio (**b**), nuclear length/width ratio (**c**), and nuclear positioning (**e**) in shControl and shAmotL2 treated HAoECs under static conditions. n = 158 (in **b,c**) and n = 75 (in **e**) were quantified in shControl group, and n = 129 (in **b,c**) and n = 68 (in **e**) in shAmotL2 group, **d**, Representative images of nuclear morphology (Hoechst, blue) under both static and flow (14 dyn/cm^2^, 48 h) conditions. The quantitative analysis of cellular orientation (n = 100 cells from three independent experiments) is shown in polar bar charts. Scale bars: 20 µm. **f**, WB analysis of active Rho/whole Rho input. Data was gathered from three independent experiments. *n.s*., not significant. Source data

Next, we investigated whether AmotL2 is required for arterial endothelial compliance to laminar flow in vitro. For this purpose, we used a short hairpin lentiviral approach to deplete AmotL2 in human aortic ECs (HAoECs; Extended Data Fig. 3a). No differences between control and AmotL2-depleted cells in cellular and nuclear shape were detectable under static conditions (Fig. 3a and Extended Data Fig. 3b,c). To recapitulate arterial flow conditions, cells were exposed to 14 dynes per cm^2^ for 48 h in a flow chamber using an ibidi pump system. Control HAoECs exhibited an elongated phenotype and aligned in the direction of flow; however, the depletion of AmotL2 resulted in failure to elongate and align (Fig. 3a–c). We could further show that AmotL2 was required for controlling nuclear shape, orientation and positioning. Consistent with the cellular shape change, nuclei also exhibited a rounder shape and could not align in the direction of flow; there was also a lack of positioning at the centre of the cells when compared to control HAoECs (Fig. 3d,e and Extended Data Fig. 3d,e).

As shown in Fig. 3f, exposure to laminar shear stress not only aligned cells with flow but also triggered the formation of radial actin filaments. These actin fibres mechanically connected cells via VE-cadherin and terminate or overlap with the cellular nucleus. Interestingly, even though the total amount of actin remained the same, perturbed actin filaments were excluded from the nuclear area, resulting in a relatively actin-free peri-nuclear zone (Fig. 3f,g). The Rho family are key regulators of the actin cytoskeleton^29^. Interestingly, depletion of AmotL2 was consistent with decreased levels of active Rho (Fig. 3h and Extended Data Fig. 3f)

### AmotL2 couples VE-cadherin to the nuclear lamina

Actin filaments are coupled to the nuclear membrane through the LINC complex^28,30–32^. This complex consisting of SUN domain proteins (SUN1 and SUN2) and KASH domain proteins (Nesprin-2) connects to Lamin A/C of the nuclear membrane. Therefore, we next investigated a possible connection among VE-cadherin, AmotL2, actin and the LINC complex.

**Fig. 4:**
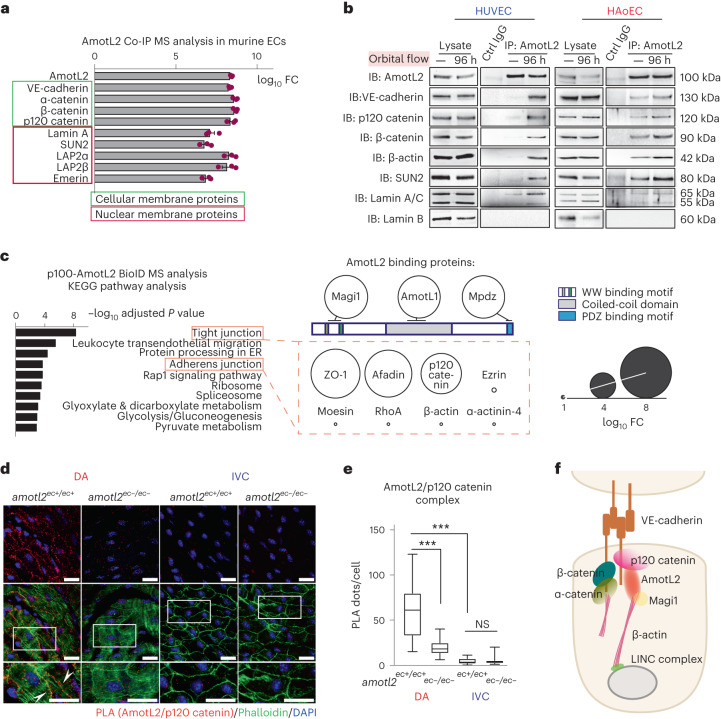
Identification of the AmotL2 adhesome. **a**, Cellular membrane proteins (framed in green box) and nuclear membrane proteins (framed in red box) were identified from AmotL2 immunoprecipitates in MS1 cells by MS analysis (1,670 proteins in total). The data are displayed with log_10_ fold change (FC) as compared to control IP samples. Data were obtained from three independent experiments and are presented as mean ± s.e.m. **b**, Western Blot (WB) analysis of AmotL2 IP samples in HUVECs and HAoECs, with or without 96-h after orbital shaking (300 r.p.m.). The cells growing at the periphery (6 cm to the edge of a 15-cm dish) with alignment were harvested. **c**, The top 10 KEGG pathways enriched among proteins (*n* = 121) biotinylated by BirA-p100-AmotL2 construct as identified by MS analysis in MS1 cells. Samples of each group were from three independent experiments. Proteins of interest are shown in circles. The circles’ sizes indicate the log_10_ FC values. The known direct binders are displayed next to the corresponding binding domains of p100-AmotL2. New protein binders belonging to the ‘tight junction’ and ‘adherens junction’ KEGG pathway are presented in the orange box. **d**, Whole-mount staining of *amotl2*^*ec+/ec+*^ and *amotl2*^*ec*−*/ec*−^ (male, 3 months of age) in DA and IVC with PLA signals (AmotL2/p120 catenin) in red and phalloidin in green. The boxed area is magnified below. Three experiments were performed. Scale bars, 20 µm. **e**, Quantification of PLA signals per cell in DA and IVC of *amotl2*^*ec+/ec+*^ and *amotl2*^*ec*−*/ec*−^ mice shown in box plot. Eighty-one (86) cells from three mice were quantified in *amotl2*^*ec+/ec+*^ (*amotl2*^*ec*−*/ec*−^) DAs. Eighty-two (91) cells from three mice in *amotl2*^*ec+/ec+*^ (*amotl2*^*ec*−*/ec*−^) IVCs. Staining was performed three times. ^***^P < 0.001. **f**, Hypothetical schematic of AmotL2-associated mechanoresponsive complex. ER, endoplasmic reticulum. Source data

**Extended Data Fig. 4:**
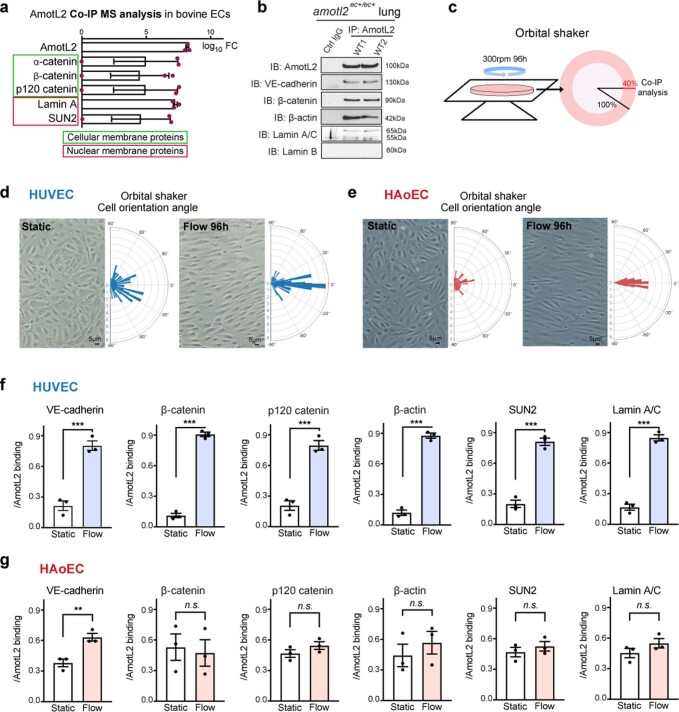
AmotL2 couples VE-cadherin to the LINC complex. **a**, Catenins in cellular membrane proteins (framed in the green box) and nuclear membrane proteins (framed in the red box) were identified from AmotL2 co-IP in BAE cells by MS analysis (325 proteins in total). The data was displayed with log_10_ FC as compared to control IP samples, mean ± s.e.m. Samples from three independent experiments were gathered for the analysis. **b**, Mouse lung tissues from two randomly picked *amotl2*^*ec+/ec+*^ mice were lysed, subjected to IP with rabbit IgG or AmotL2 antibody and analysed by WB. The mice were aged 7–9 months. Similar results were obtained from two other independent experiments. **c**, Schematic description how circulatory flow was applied to HUVECs and HAoECs cultured in 15 cm dish (300 r.p.m., 96 h). The periphery area with width of 6 cm (pink area) is where the cells were harvested for co-IP experiments. Bright field images of HUVECs (**d**) and HAoECs (**e**) located in the pink area illustrated the cell morphology in both static and post-flow conditions (96 h on orbital shaker). Scale bars: 5 µm. Quantitative analysis of cellular orientation (n = 137 HUVEC; n = 72 HAoEC) is shown in polar bar charts. The amount of AmotL2 binding proteins with or without 96 h orbital flow were quantified in HUVECs (**f**) and HAoECs (**g**). The data were gathered from WB analyses from three independent AmotL2 co-IP experiments for both HUVECs and HAoECs. Data are presented as mean ± s.e.m. Source data

We used a co-immunoprecipitation (co-IP) analysis approach to identify AmotL2-associated immunocomplexes from both murine endothelial cell line (MS1) and primary bovine aortic endothelial (BAE) cells. By mass spectrometry (MS) analysis, we identified cellular membrane protein VE-cadherin and α-catenin, β-catenin and p120 catenin as well as nuclear laminal proteins, such as SUN2, Emerin, LAP2α, LAP2β and Lamin A (Fig. 4a, Extended Data Fig. 4a and Supplementary Tables 1 and 2). Mouse lungs consist of approximately 10–20% ECs. We performed IP using AmotL2 antibodies and could verify that AmotL2 associated with VE-cadherin, β-catenin, β-actin and Lamin A also in vivo (Extended Data Fig. 4b).

The higher expression levels of AmotL2 detected in the DA as compared to the IVC raised the question of whether AmotL2-associated complex is formed when exposed to shear stress. We plated primary human umbilical venous ECs (HUVECs) or HAoECs in a 15-cm culture dish on an orbital shaker to analyse formation of the VE-cadherin/AmotL2 in response to flow (schematic in Extended Data Fig. 4c). As previously described^33^, the alignment of the cells at the periphery area was observed after 96 h (Extended Data Fig. 4d,e). The co-IP experiments showed that the VE-cadherin/AmotL2 complex was formed upon exposure to shear stress in HUVECs (Fig. 4b and Extended Data Fig. 4f). However, the connection between AmotL2 and cellular and nuclear membranes in HAoECs existed regardless of flow (Fig. 4b and Extended Data Fig. 4g).

**Extended Data Fig. 5:**
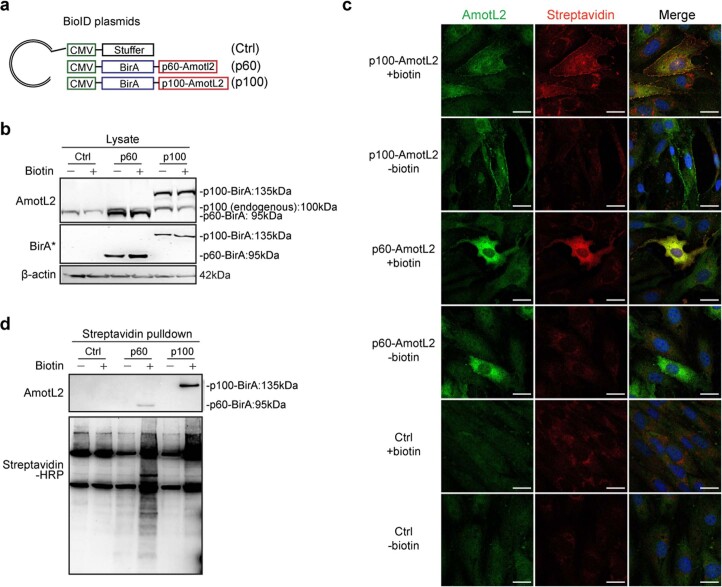
BioID analysis of the AmotL2 adhesome. **a**, Schematic illustrating the BioID plasmids used for lentivirus particle construction and subsequent transfection for BioID experiments. **b**, WB analysis of BioID-fusion protein expression in transfected MS1 cells, including empty vector, p60-AmotL2 BioID and p100-AmotL2 BioID. Biotin was added for 16 h to trigger the biotinylating. Molecular weights of the endogenous and fused proteins are indicated to the right of the blot. **c**, IF staining of AmotL2 (green) and streptavidin (red) in transfected MS1 cells. Nuclear DNA was stained with DAPI (blue) in the merged images. Scale bars: 20 µm. **d**, WB analysis of streptavidin Sepharose beads pulled-down, probed with antibodies against AmotL2 and streptavidin-HRP. **b-d**, BioID samples were gathered from three independent experiments. and the validation (**b-d)** have been performed in parallel. Source data

Next, we investigated how AmotL2 associated with the VE-cadherin complex. We made use of the proximity-dependent biotin identification (BioID) technique, which measures protein–protein interaction in living cells^34^. Proteins that are within 20 nm are biotinylated by a biotin ligase. Biotinylated proteins are then purified and identified by MS. For this purpose, we fused the biotin ligase gene (BirA) to the N-terminal part of the p100-AmotL2 DNA sequence. As a control, we used a construct with an N-terminal deletion of 370 amino acids (Extended Data Fig. 5a). The constructs were stably transfected into MS1 murine ECs. Expression levels were analysed by western blot and were similar to that of endogenous protein (Extended Data Fig. 5b). Immunofluorescence (IF) staining further showed that BirA-p100-AmotL2 localized to cellular junctions, whereas the truncated p60-AmotL2 protein was expressed in the cytoplasm (Extended Data Fig. 5c).

Purified biotinylated proteins (Extended Data Fig. 5d) were analysed by MS, resulting in the identification of 121 candidate interactors. Known direct binders to AmotL2, such as AmotL1, Magi1 and Mpdz, were part of the list of proteins identified (Fig. 4c and Supplementary Table 3). KEGG pathway analysis showed enrichment of proteins related to tight junction and adherens junction (Fig. 4c). These junctional proteins included ZO-1, Afadin and p120 catenin. p120 catenin binds directly to the submembrane domain of VE-cadherin, which is distinct from the β-catenin interaction site and may, therefore, indirectly couple AmotL2 to the junctional complex^35^.

Next, we assessed whether AmotL2 and p120 catenin formed a complex in ECs in vivo. For this purpose, we used the Proximity Ligation Assay (PLA), which detects proteins that are within 40 nm of each other^36^. Distinct complex formation was detected in ECs of wild-type (WT) DA as compared to ECs of *amotl2*^*ec−/ec*−^ DA or IVC (Fig. 4d,e).

Taken together, our data suggest a model where junctional VE-cadherin/p120 catenin/AmotL2 are associated with actin filaments that connect to the nuclear lamina (schematic in Fig. 4f).

### Deletion of AmotL2 promotes vascular inflammation in vivo

**Fig. 5:**
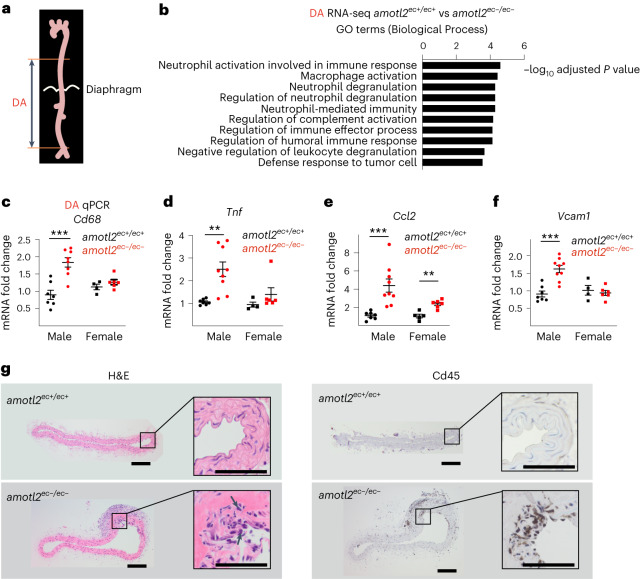
Endothelial-specific AmotL2 deletion promotes aortic wall inflammation. **a**, Schematic of the full-length aorta. The samples used for transcriptome analysis were from the DA (including part of thoracic aorta and whole abdominal aortas). **b**, mRNA isolated from DAs of *amotl2*^ec+/ec+^ (*n* = 3) and *amotl2*^ec−/ec−^ (*n* = 5) mice aged 7–9 months were sent for RNA-seq analysis. In total, 63 significantly differentially expressed genes—that is, with adjusted *P* value (*P*_adj_) < 0.05—were subjected to GO term matching. Enriched GO terms are listed in the graph according to the −log_10_ *P*_adj_. **c**–**f**, Relative mRNA expression levels of *Cd68*, *Tnf*, *Ccl2* and *Vcam1* using TaqMan probe-based qPCR. Total mRNA was isolated from DA tissues of approximately 13 *amotl2*^ec+/ec+^ mice in black dots (male *n* = 7 in **c**–**f**; female *n* = 4 in **c**,**d**,**f** and *n* = 5 in **e**) and 15 *amotl2*^ec−/ec−^ mice in red dots (male *n* = 8 in **c** and *n* = 9 in **d**,**e**,**f**; female *n* = 6 in **c**–**f**). Relative expression levels were normalized to male *amotl2*^ec+/ec+^ mice. Fold changes are shown as mean ± s.e.m. ^***^P < 0.001. The unlabelled comparisons indicate no statistical significance. **g**, Immunohistochemistry images of the DAs from *amotl2*^ec+/ec+^ and *amotl2*^ec−/ec−^ mice. Hematoxylin and eosin (H&E) (left) and Cd45 (middle) staining was performed on the paraffin-embedded cross-sections. The boxed area is magnified and placed to the right. Cross-section from three DAs in *amotl2*^ec+/ec+^ mice and two aneurysms in *amotl2*^ec−/ec−^ mice were performed with immunohistochemical staining. Scale bars, 100 µm. Source data

EC alignment and cytoskeletal reorganization in response to laminar blood flow is protective against inflammation^3^. The lack of alignment of *amotl2*^*ec*−*/ec*−^ EC in the aorta raised the question of whether this was accompanied by a pro-inflammatory response. mRNA was, therefore, isolated from DAs in both *amotl2*^*ec+/ec+*^ (*n* = 3) and *amotl2*^*ec*−*/ec*−^ (*n* = 5) mice and analysed by RNA sequencing (RNA-seq) (Fig. 5a). Due to high variability among individual mice, only 63 genes were identified to be differentially expressed (adjusted *P* < 0.05) between *amotl2*^*ec+/ec+*^ and *amotl2*^*ec*−*/ec*−^ groups. However, those genes were enriched in immuno-related Gene Ontology (GO) terms, such as ‘Neutrophil activation involved in immune response’ and ‘macrophage activation’ (Fig. 5b and Supplementary Table 4).

**Extended Data Fig. 6:**
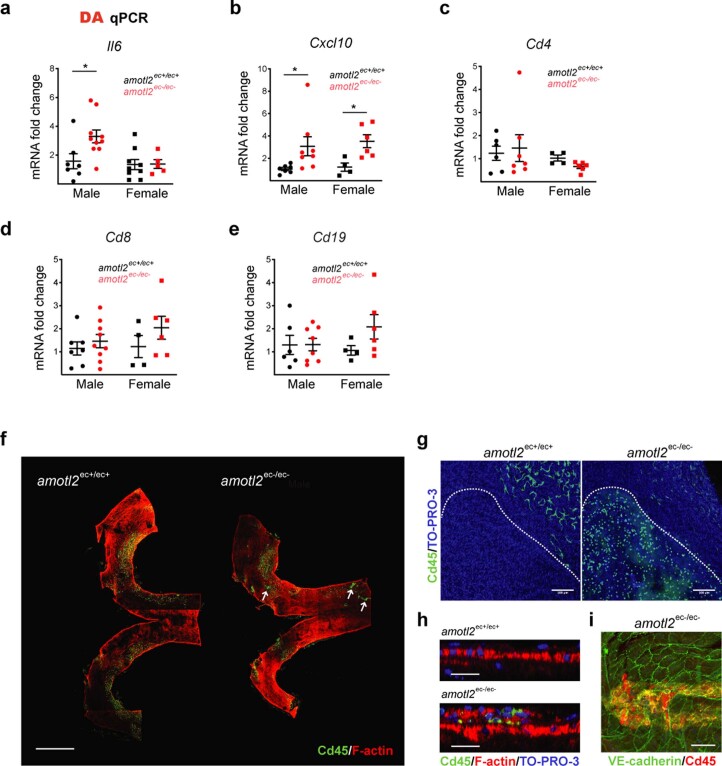
Induction of inflammatory genes in the *amotl2*^*ec*−*/ec*−^ mice. **a-e**, mRNA was isolated separately from DA of mice (aged 7–9 months) and analysed by TaqMan qRT-PCR. Relative expression levels of *Il6* (**a**), *Cxcl10* (**b**), *Cd4* (**c**), *Cd8* (**d**), and *Cd19* (**e**) were normalized to *amotl2*^*ec+/ec+*^ male mice. Samples were obtained from approx. 10 *amotl2*^*ec+/ec+*^ mice in black dots (Male n = 7 in **a,b,d**; n = 6 in **c,e**. Female n = 9 in **a**; n = 4 in **b,c,d,e**) and 14 *amotl2*^*ec*−*/ec*−^ mice in red dots (Male n = 10 in **a**; n = 8 in **b,e**; n = 7 in **c**; n = 9 in **d**. Female n = 5 in **a**; n = 6 in **b,c,d,e**). FCs were shown as mean ± s.e.m. ^*^P < 0.05. All unlabelled statistical analyses in the graphs indicate “not statistically significant”. **f**, Whole-mount staining of aortic arches of *amotl2*^*ec+/ec+*^ and *amotl2*^*ec*−*/ec*−^ mice, stained with Cd45 (green) and phalloidin (red). The white arrows point at the clusters of Cd45 positive cells, which indicates the endothelial lesions. **g**, Representative images of Cd45 positive cell clusters (green) in *amotl2*^*ec*−*/ec*−^ aortic arch (nine out of 20) within the area outlined by the white dashed line. These were not present in *amotl2*^*ec+/ec+*^ arch (n = 20). **h**, Orthogonal view of *amotl2*^*ec+/ec+*^ arch and *amotl2*^*ec*−*/ec*−^ lesion area stained with Cd45 (green), phalloidin (red) and TO-PRO-3 (blue). The luminal cells are on the top layer in the images. **i**, 3D-projection view of Cd45^+^ (red) cells invading the endothelium (green) in *amotl2*^*ec*−*/ec*−^ arch. The image was processed in ImageJ. **f-i**, 20 arches in each group have been stained and Cd45 enriched area were found in nine of them. Scale bars: (**f**) 1000 µm, (**g**) 100 µm, and (**h** and **i**) 20 µm. Source data

The mRNA expression of the identified inflammation-associated genes was verified by TaqMan qRT–PCR. Interestingly, *CD68*, which is a monocyte lineage marker, was preferentially upregulated in male AmotL2-deficient mice (Fig. 5c). Similar findings were observed with the cytokines *Tnf*, *Ccl2*, *Il6* and *Cxcl10* (Fig. 5d,e and Extended Data Fig. 6a,b). Expression levels of the T cell and B cell markers *Cd4*, *Cd8* and *Cd19* showed no significant difference when comparing WT and AmotL2-deficient mice (Extended Data Fig. 6c–e). Consistent with the upregulation of cytokines, vascular cell adhesion protein 1 (*Vcam1*), which mediates monocyte adhesion to the endothelium, was also upregulated in male *amotl2*^*ec*−*/ec*−^ mice (Fig. 5f).

Next, we performed immunostaining of the descending aorta to analyse whether the upregulation of inflammatory markers also corresponded to the presence of inflammatory cells. Cd45^+^ cells were detected in the sub-renal area of the descending aorta as analysed by immunohistochemistry (Fig. 5g). Monocyte infiltration was also observed in the outer curvature of the aortic arch, which differed from the spindle-like Cd45^+^ macrophages that reside in the inner curvature as well as in arterial bifurcations (Extended Data Fig. 6f–i).

### AmotL2 supresses expression of pro-inflammatory genes

**Fig. 6:**
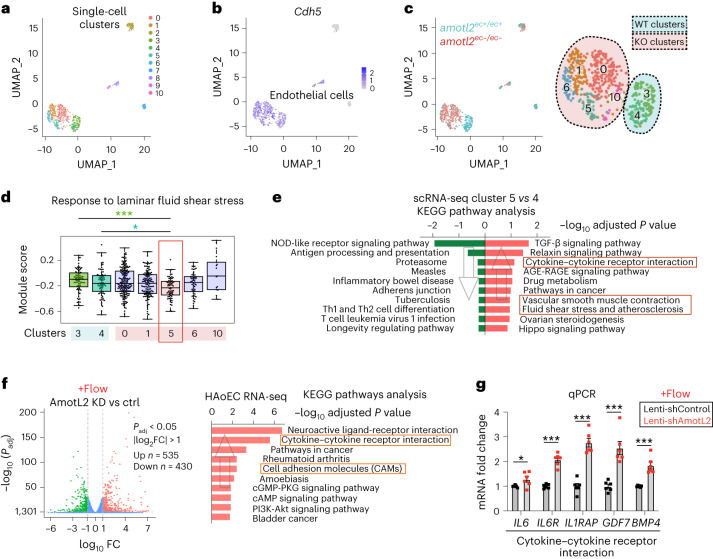
AmotL2 depletion promotes an inflammatory phenotype of ECs. **a**, Uniform manifold approximation and projection (UMAP) plot showing the distribution of single-cell transcriptomes from single aortic cells (Cd31^+^Cd45^−^) purified from *amotl2*^*ec+/ec+*^ and *amotl2*^*ec*−*/ec*−^ (*n* = 6 for each group from two independent experiments) mice aged 3 months. The 11 clusters identified were colour coded. **b**, The expression of *Cdh5* marks the EC clusters in purple. **c**, Single cells from *amotl2*^*ec+/ec+*^ and *amotl2*^*ec*−*/ec*−^ DA are indicated in blue and pink dots on the left diagram. Major endothelial clusters are magnified, and ‘WT clusters’ (*n* = 2) and ‘KO clusters’ (*n* = 5) are indicated with blue and pink backgrounds, respectively. **d**, Beeswarm box plots showing GO term ‘response to laminar fluid shear stress’ (GO: 00346616) enriched in each cluster. Data were gathered from six mice for each group from two independent experiments. **e**, Top 10 enriched KEGG pathways ranked by –log_10_ adjusted *P* (*P*_adj_) when comparing cluster 5 versus cluster 4. In total, 64 upregulated and 84 downregulated genes were subjected to Enrichr (*P*_adj_ < 0.05 and log_2_ fold change (FC) > 1). The directions of the arrows indicate upregulated or downregulated pathways. **f**, Volcano plot showing differentially expressed genes (*P*_adj_ < 0.05 and log_2_ FC > 1) between HAoECs treated with scrambled control and AmotL2 lenti-shRNA under flow condition (14 dynes per cm^2^, 48 h). The numbers of significantly upregulated and downregulated genes are indicated in the graph. **g**, mRNA expression of the genes from ‘cytokine–cytokine receptor interaction’ using SYBR Green primers (normalized to control HAoEC under static condition). FC is presented as mean ± s.e.m. Samples were gathered from three independent experiments. ^*^P < 0.05 and ^***^P < 0.001. ctrl, control; KD, knockdown. Source data

**Extended Data Fig. 7:**
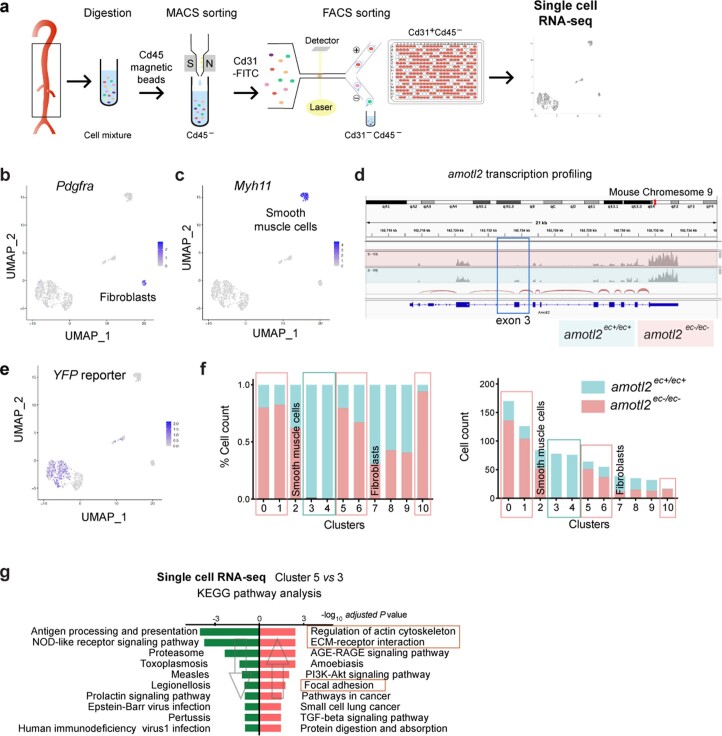
EC clustered in *amotl2*^*ec*−*/ec−*^ DA identified by scRNA-seq. **a**, Schematic workflow of the scRNA-seq experimental strategy. *amotl2*^*ec+/ec+*^ (n = 6 from two independent experiments) and *amotl2*^*ec*−*/ec*−^ mice (n = 6 from two independent experiments) aortas were dissected and enzymatically digested for single cell suspension. Cd45^−^ cells were negatively isolated by Cd45 magnetic beads and Cd31^+^Cd45^−^ cells were sorted by FACS. **b-c**, The expression of specific markers for fibroblasts (*Pdgfra*) and smooth muscle cells (*myh11*) in the UMAP. **d**, *amotl2* transcription profiling at mouse chromosome 9 of *amotl2*^*ec+/ec+*^ (blue background) and *amotl2*^*ec*−*/ec*−^ (pink background) mice. The *amotl2* floxed sites are in exon 3 labelled in the blue frame. **e**, Expression of *YFP* reporter, the marker for Cre-recombinase, indicating the activation of *amotl2*^*ec*−*/ec*−^, coded in purple. **f**, Bar graphs displaying the cell distribution of *amotl2*^*ec+/ec+*^ and *amotl2*^*ec*−*/ec*−^ mice (blue and pink backgrounds, respectively) across all cell clusters, by percentage of the whole cell count (left panel) and cell number (right panel). The majority of ECs in cluster 3 and 4 (blue frames) were derived from *amotl2*^*ec+/ec+*^ mice, whereas most cells in cluster 0, 1, 5, 6, and 10 (in pink frames) are from *amotl2*^*ec*−*/ec*−^ mice. **g**, Top 10 enriched KEGG pathways ranked by –log_10_ *padj* when comparing cluster 5 vs. cluster 3. In total, 26 up-regulated and 50 down-regulated genes were subjected to Enrichr (*padj* < 0.05 and *log*_*2*_ *FC* > 1). ECM-relevant pathways are highlighted by red frames. Source data

We went on to assess the phenotypic changes occurring specifically in the aortic EC after AmotL2 depletion. For this aim, the luminal wall of mouse aortas was digested with collagenase, and ECs were purified by negatively depleting Cd45^+^ cells before fluorescence-activated cell sorting (FACS) for Cd31 positivity. The isolated cells were then subjected to single-cell RNA sequencing (scRNA-seq) analysis (Extended Data Fig. 7a). On average, around 9,000 genes were detected per cell. Out of 11 distinct clusters identified by unsupervised classification (Fig. 6a), nine were classified as ECs based on expression of VE-cadherin (*Cdh5*), whereas the remaining clusters were identified as smooth muscle cells and fibroblasts (Fig. 6b and Extended Data Fig. 7b,c). Recombination of AmotL2 in ECs from *amotl2*^*ec*−*/ec*−^ mice was verified by exon 3 deletion in *amotl2* transcriptome profile and positive expression of *YFP* reporter (Extended Data Fig. 7d,e). ECs were largely separated by *amotl2* status, but there was considerable heterogeneity within both WT and KO cells, which organized into two and five clusters, respectively (Fig. 6c and Extended Data Fig. 7f). This heterogeneity is likely of functional significance, because subclusters showed clear differences in their expression of gene sets related to shear stress. In particular, we found that cluster 5, specific to KO mice, expressed lower level in genes related to laminar fluid shear stress (Fig. 6d), when compared to either of the WT-specific clusters. Further KEGG pathway analysis of this cluster compared to the main WT clusters revealed enrichment of genes involved in ‘Cytokine–cytokine receptor interaction’ and ‘Regulation of actin cytoskeleton’ (Fig. 6e, Extended Data Fig. 7g and Supplementary Tables 5 and 6). Thus, our data suggest that AmotL2 deletion affects ECs in a subtype-specific manner and that a subset of the ECs is likely responsible for the AmotL2 deletion phenotype.

**Extended Data Fig. 8:**
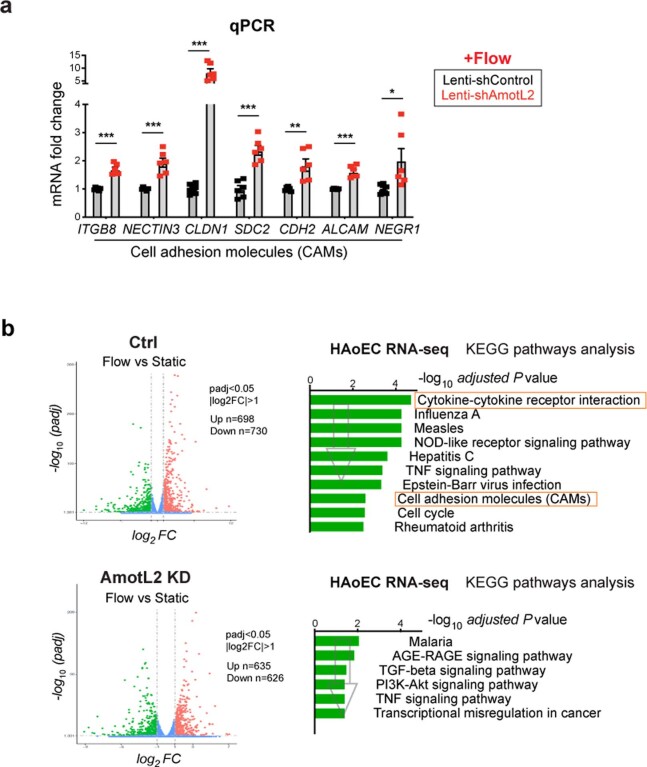
AmotL2-depletion induces a pro-inflammatory phenotype in HAoECs. **a**, Relative mRNA expression of genes from KEGG pathway ‘Cell adhesion molecules (CAMs)’ were determined with SYBR green primers-based qPCR. FCs were normalized to the level of control HAoECs after 48-h flow (n = 6 for each group from three independent experiments). FC is presented as mean ± s.e.m. **b**, Top 10 down-regulated KEGG pathways influenced by flow compared to static conditions on scramble lenti-shRNA (upper panel) and AmotL2 lenti-shRNA (lower panel)-treated HAoECs (n = 2 for each group from one experiment). For all the volcano plots, the numbers of up-and down-regulated genes and the cut-offs for analyses are displayed by the graphs. ^*^P < 0.05. ^**^P < 0.01. ^***^P < 0.001. Source data

In Fig. 3a, we demonstrated that AmotL2 is necessary for in vitro endothelial alignment in response to laminar flow. After this, we investigated if a lack of alignment due to AmotL2 depletion would impact the expression of pro-inflammatory genes. We analysed control and shAmotL2-depleted cells under static and flow conditions and gene expression profiles subsequently identified by RNA-seq. After exposing the cells to laminar flow for 48 h, our RNA-seq analysis revealed an upregulation of the KEGG pathways ‘Cytokine–cytokine receptor interaction’ and ‘Cell adhesion molecules (CAMs)’ in shAmotL2-treated cells (Fig. 6f and Supplementary Table 7). We further confirmed the induction of key inflammatory genes, such as *IL6*, *IL6R*, *IL1RAP*, *GDF7* and *BMP4*, through qPCR (Fig. 6g, Extended Data Fig. 8a,b and Supplementary Table 8).

### AmotL2 depletion promotes aneurysm formation in male mice

**Fig. 7:**
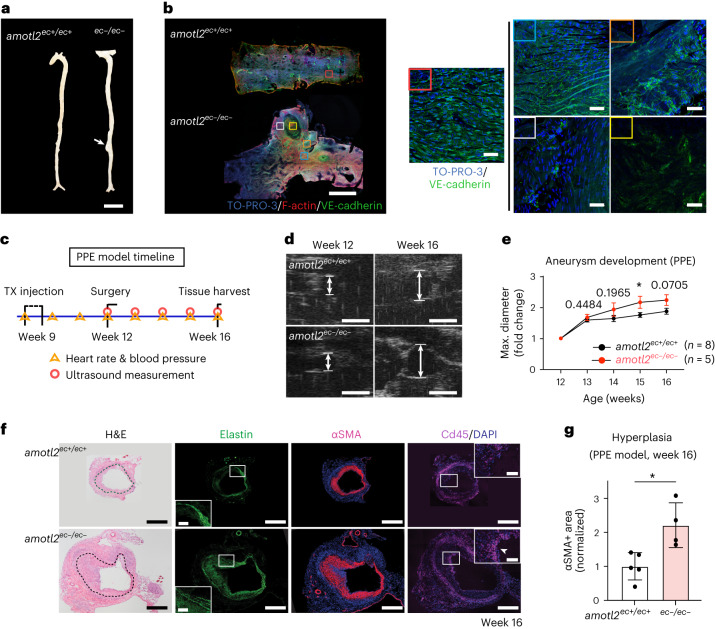
Gender-specific formation of aneurysms in amotl2^ec−/ec−^ mice. **a**, The representative dissected full-length aorta from *amotl2*^*ec+/ec+*^ and *amotl2*^*ec*−*/ec*−^ mice (aged 7–9 months). White arrow indicates the AAA, which occurred above the renal artery bifurcations. **b**, Whole-mount staining (VE-cadherin in green, phalloidin in red and TO-PRO-3 in blue) on *amotl2*^*ec+/ec+*^ DA and aneurysm in *amotl2*^*ec*−*/ec*−^ mice. The selective area (boxed) is magnified and presented in the right panel. The red box shows WT EC morphology in *amotl2*^*ec+/ec+*^ DA. The aneurysm progress was followed by the magnified area (in blue-orange-white-yellow box). One (out of five) aneurysm from *amotl2*^*ec*−*/ec*−^ mice was examined in whole-mount staining. **c**, The timeline of tamoxifen injections and PPE surgery to induce an aneurysm in *amotl2*^*flox/flox*^ Cdh5(PAC)^CreERT2^ ROSA26-EYFP mice. Only male mice were included. Hemodynamic parameters were examined once a week. Heart rate and blood pressure were measured before ultrasound tests. **d**, Ultrasound images captured during ultrasound examination before surgery (week 12) and 28 d after surgery (week 16). Arrows illustrate how the aortic diameter was determined. Eight *amotl2*^*ec+/ec+*^ and five *amotl2*^*ec*−*/ec*−^ mice were examined. **e**, The line graph displaying the maximum aortic diameter throughout the course of the experiment (*n* = 8 for *amotl2*^*ec+/ec+*^ and *n* = 5 for *amotl2*^*ec*−*/ec*−^ mice) by ultrasound. Data are presented as mean ± s.e.m. ^*^P < 0.05. **f**, Cryosection of the aneurysm from *amotl2*^*ec+/ec+*^ and *amotl2*^*ec*−*/ec*−^ mice stained with hematoxylin and eosin (H&E), αSMA and Cd45 as well as elastin. Boxed areas are magnified next to the images. White arrows indicate the Cd45^+^ cluster in *amotl2*^*ec*−*/ec*−^ mice. Five *amotl2*^*ec+/ec+*^ and four *amotl2*^*ec*−*/ec*−^ mice were examined. **g**, Quantification of the area of αSMA^+^ staining shown in box plots (*n* = 5 for *amotl2*^*ec+/ec+*^ and *n* = 4 for *amotl2*^*ec*−*/ec*−^ mice). Scale bars: 1,000 µm (**a**); (**b**) left panel: 1,000 µm and right panel (five images): 25 µm; 1 mm (**d**); and 250 µm (**f**) in all graphs except 10 µm in the magnified images with white frames. TX, tamoxifen. Source data

**Extended Data Fig. 9:**
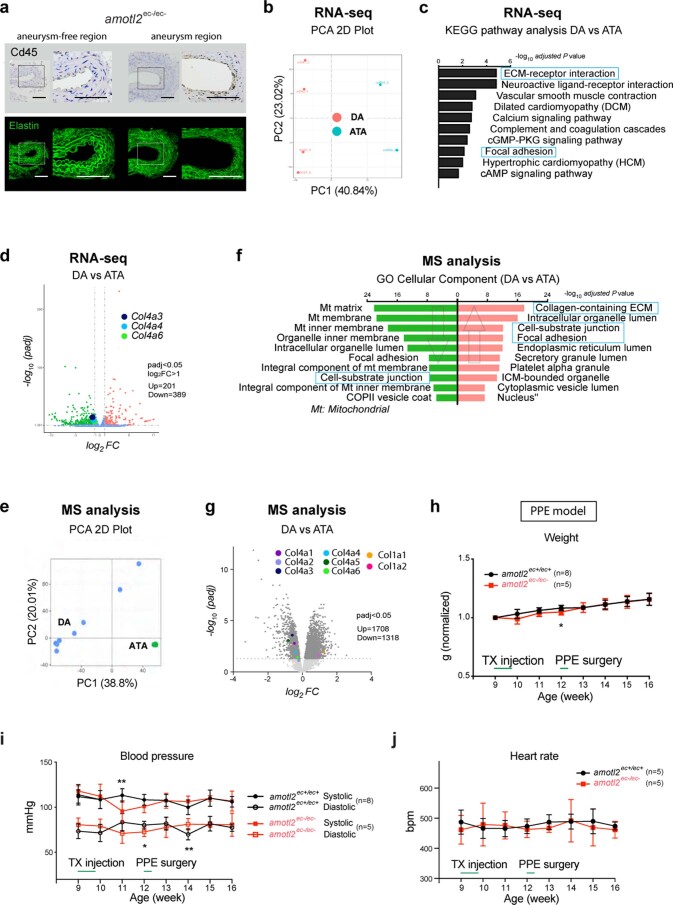
Analysis of *amotl2*^*ec*−*/ec*−^ induced AAA and gene and protein expression analyses of ascending vs. descending aorta. **a**, Images of DAs from *amotl2*^*ec*−*/ec*−^ mice with Cd45 staining and elastin autofluorescence. Regions with and without aneurysms are presented and the boxed areas are magnified on the right. Two (out of five) aneurysms from male *amotl2*^*ec*−*/ec*−^ mice were examined. Scale bars: 50 µm. **b**, The PCA 2D plot showing the distributed transcriptome profiles of DA and Ascending Thoracic Aorta (ATA) samples. All tissues were from *amotl2*^*ec+/ec+*^ mice (aged eight months). mRNA isolated from DA (n = 4, pooled from eight mice) and ATA (n = 2, pooled from eight mice) were analysed by RNA-seq. **c**, The bar diagram showing the top 10 KEGG pathways enriched in DA gene signatures compared to ATA. 201 up-regulated and 389 down-regulated genes were pooled together and subjected in Enrichr for the analyses (*padj* < 0.05 and *log*_*2*_ *FC* > 1). **d**, The volcano plot displaying differentially expressed genes between DA and ATA (*padj* < 0.05 and *log*_*2*_ *FC* > 1). 201 genes were up-regulated (red dots) and 389 down-regulated (blue dots). C*ol4a-3*, *-4*, and *-6* are labelled in dark blue, blue, and green. **e**, The PCA 2D plot showing the distributed protein profiling of DA and ATA in *amotl2*^*ec+/ec+*^ mice (aged 8 months). Protein isolated from DA (n = 8, from eight mice) and ATA (n = 3, pooled from six mice) were used for MS analysis. **f**, Top 10 enriched GO terms (Cellular Component) on protein profile (*padj* < 0.05) in DA compared to ATA. Analyses were performed in both up-regulated proteins (red, n = 1708) and down-regulated proteins (green, n = 1318). ECM related pathways are highlighted in blue frames. **g**, Volcano plot illustrating the significantly differentially expressed proteins (grey dots above the dashed line, *padj* < 0.05) in DA comparing to ATA. Col4a1-6, Col1a1 and Col1a2 are indicated in different colour codes. Aortic samples in (**b-j**) were harvested in four independent time points. **h-j**, Line graphs displaying weight change (**h**), blood pressure (**i**) and heart rate (**j**) through the course of the PPE experiment (n = 5-8 in *amotl2*^*ec+/ec+*^ mice and n = 5 in *amotl2*^*ec*−*/ec*−^ mice). Data are presented as mean ± s.d. ^*^P < 0.05. ^**^P < 0.01. There was no statistical significance detected in the rest time points. Source data

Aortic inflammation is associated with the development of arterial aneurysm^37^. As shown in Fig. 7a, spontaneous formation of AAA (dilatation > 1.5 times normal size) in the proximity of the renal arterial branch was detected in *amotl2*^*ec*−*/ec*−^ mice but not in the ascending or descending thoracic aortas. Interestingly, 20% of male *amotl2*^*ec*−*/ec*−^ (5/25) mice developed an AAA; however, no aneurysm was detected in females (0/20) or in *amotl2*^*ec+/ec+*^ mice (36 mice: 20 male and 16 female). Imaging analysis of a typical AAA revealed damage to the endothelium as well as the vessel wall (Fig. 7b). Cross-sections of AAAs further showed degradation of elastin fibres as well as infiltration of Cd45^+^ cells (Extended Data Fig. 9a).

We observed monocyte infiltration in both the aortic arch as well as in the descending aorta; however, aneurysms were formed only in the latter. To understand this apparent discrepancy, we compared gene and protein expression in the two aortic locations. Profiling of the ascending thoracic aorta (ATA) versus DA showed a difference in expression of genes involved in ‘ECM receptor interaction’ as well as ‘Focal adhesion’ (Extended Data Fig. 9b–d and Supplementary Table 9). Moreover, we also processed the DA and ATA tissues for protein profiling by MS analysis. Interestingly, the results were very consistent with transcriptome analysis, in that ECM proteins, such as collagen I and collagen IV, were differentially expressed in DA as compared to the ascending aorta (Extended Data Fig. 9e–g and Supplementary Table 10). The lower amount of collagen IV in the descending aorta may explain the sensitivity to aneurysm formation as hemizygosity of *Col4a1/a2* augments AAA formation in mice^38^.

Interestingly, the aneurysms observed in male *amotl2*^*ec*−*/ec*−^ mice were formed spontaneously 1 month after gene deletion without any changes in diet, blood pressure or other insults. Next, we wanted to study the influence of AmotL2 deletion in an established murine model of aneurysm formation. To this end, we used the periadventitial porcine pancreatic elastase (PPE) model that is based on local elastase bathing to weaken the vessel wall and, thereby, induce aneurysm formation^39,40^. Ablation of AmotL2 was induced in 8-week-old male mice, followed by surgery and local elastase exposure at week 12 (experimental setup shown in Fig. 7c). The progression of aneurysm formation was followed weekly by ultrasound imaging (Fig. 7d,e). No significant change was observed in weight gain or hemodynamic parameters, such as heart rate or blood pressure, between the two groups (Extended Data Fig. 9h–j). Ultrasound imaging revealed a significantly larger lumen in the *amotl2*^*ec*−*/ec*−^ mice at week 15. Immunohistochemical analysis at week 16 showed extensive intimal thickening due to expansion of α-smooth muscle actin-positive (αSMA^+^) cells (Fig. 7f,g).

### AmotL2 and inflammation in patients with AAA

**Fig. 8:**
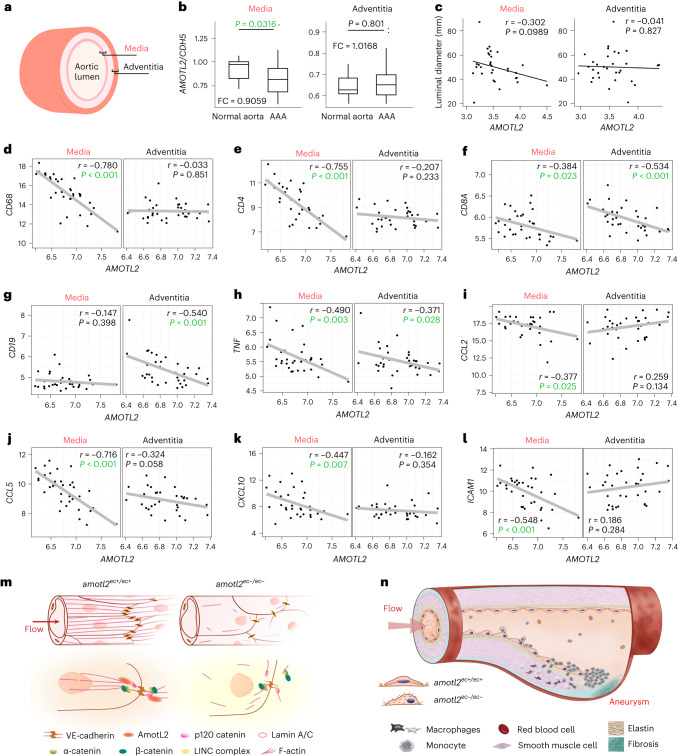
AmotL2 inversely correlates with inflammation in patients with AAA. AAA samples of the intima/media region of aortic wall not covered by an intraluminal thrombus—that is, an aneurysm wall covered by an EC layer—were obtained at open AAA surgery, and mRNA was isolated from both media and adventitia. **a**, Schematic depicting anatomical view of the media and adventitia tissue in the aorta. **b**, Quantification of *AMOTL2* mRNA expression from both intact aortas (13 healthy donors) and dilated aortas (35 patients with AAA) normalized to *CDH5*. **c**, The correlations between the luminal diameter of aneurysms and *AMOTL2* mRNA expression level in medial (left) and adventitial (right) tissues in patients with AAA (*n* = 31). *AMOTL2* expression level was based on the expression of the first exon from the 3′ end, detected by specific exon probe, which represents the full-length isoform of AMOTL2. AMOTL2 correlation with *CD68* (**d**), *CD4* (**e**), *CD8A* (**f**), *CD19* (**g**), *TNF* (**h**), *CCL2* (**i**), *CCL5* (**j**), *CXCL10* (**k**) and *ICAM1* (**l**) of mRNA expression are shown. The *AMOTL2* expression level was calculated with the mean value of every exon expression detected. For each correlation analysis, samples from both media (left) and adventitia (right) are presented. The correlation between two genes was analysed using the Pearson correlation, and Pearson correlation coefficient is referred to as *r*. *P* value and *r* (microarray analysis in patients with AAA) were calculated using R version 4.1.1. *r* and *P* values are labelled in each individual figure. Statistically significant *P* value (<0.05) is highlighted in green. Twenty-one male and 10 female patients were analysed in **c**, whereas 25 male and 10 female patients were enrolled in **d**–**l**. **m**, AmotL2-associated mechanoresponsive complex forming in *amotl2*^*ec+/ec+*^ and *amotl2*^*ec*−*/ec*−^ aorta. **n**, Hypothetical schematic of the formation of an AAA. The step-by-step formation of an AAA includes EC activation, immune cell rolling and attachment, leukocyte extravasation, macrophage differentiation, thrombus formation and, finally, lumen dilation (aneurysm). FC, fold change. Source data

**Extended Data Fig. 10:**
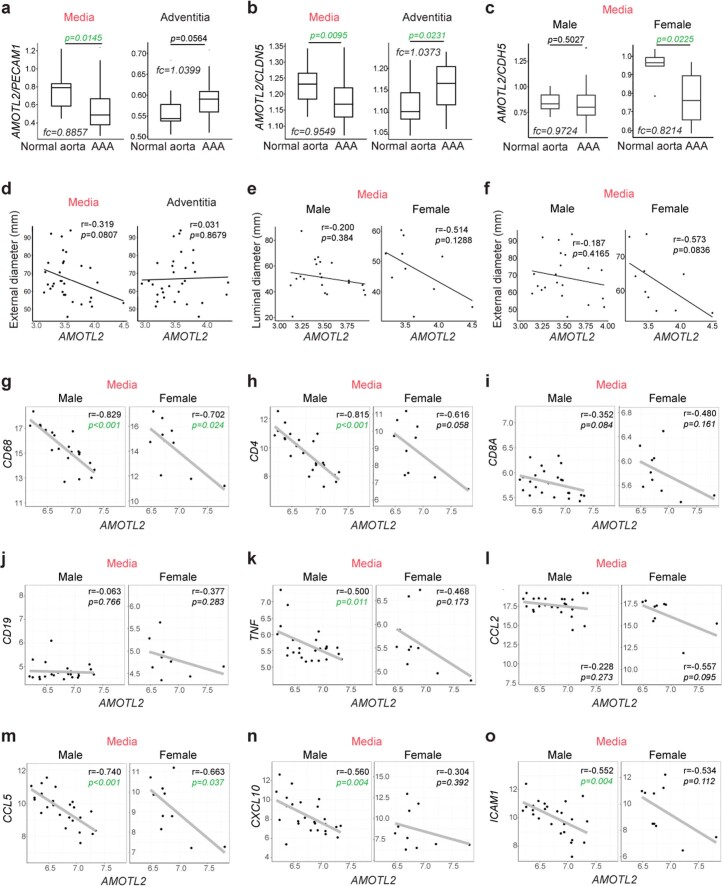
Negative correlation between AmotL2 and inflammatory markers in human AAA samples. Non-thrombotic aortic tissue (media and adventitia layer harvested separately) from AAA patients were examined. **a-b**, Quantification of *AMOTL2* mRNA expression from both intact aortae (n = 13 from healthy donors) and dilated aortae (n = 35 from AAA patients) normalized to *PECAM1* (**a**) or *CLDN5* (**b**). **c**, *AMOTL2/CDH5* expression ratio in aortic media layer was quantified in 25 male and 10 female patients separately. **d-f**, The correlations between the external diameter of aneurysms and *AMOTL2* mRNA expression level in medial (**d**, left panel) and adventitial (**d**, right panel) tissues in AAA patients (n = 31, 21 male and 10 female patients). The correlation of between both external and luminal diameters and *AMOTL2* was examined in male (**e**, n = 21) and female (**f**, n = 10) patient separately in aortic media. *AMOTL2* expression level was based on the expression of the first exon from 3’ end, detected by specific exon probe, which represents the full-length isoform of *AMOTL2*. **g-o**, *AMOTL2* correlation with *CD68* (**g**), *CD4* (**h**), *CD8A* (**i**), *CD19* (**j**), *TNF* (**k**), *CCL2* (**l**), *CCL5* (**m**), *CXCL10* (**n**), and *ICAM1*(**o**) on the mRNA level of the media layer of aortic tissue (25 male and 10 female AAA patients). AMOTL2 expression level was determined by the mean value of every exon expression detected. For each correlation analysis, samples from males are on the left and female samples are on the right. The correlation between two genes were analysed using the Pearson correlation and Pearson correlation coefficient was referred to as *r*. *P* value and *r* (Microarray analysis in AAA patients) was calculated using R version 4.1.1. *r* and the *p value* are labelled in each individual figure. Statistically significant *p value (< 0.05)* is highlighted in green. Source data

Next, we assessed whether AmotL2 gene expression was correlated with inflammation in patients with AAA. We analysed mRNA expression in surgically resected materials from both healthy donors and patients diagnosed with AAA and undergoing aneurysm repair at Karolinska University Hospital. mRNA samples were taken from both medial and adventitial layers of the intact aorta (13 donors) or AAA tissues (35 patients; Fig. 8a). *AMOTL2* expression levels, normalized to endothelial markers such as *CDH5*, were significantly lower in AAA media than in normal tissue, as shown in Fig. 8b and Extended Data Fig. 10a,b. This trend was more pronounced in females (Extended Data Fig. 10c) but was not observed in the adventitia.

Furthermore, we observed a negative correlation between *AMOTL2* expression in media and AAA maximum aortic diameter but not in adventitia. However, this was not significant according to the calculated *P* value (Fig. 8c and Extended Data Fig. 10d–f).

We also detected an inverse correlation of *AMOTL2* expression with that of monocyte/macrophage marker *CD68* as well as T cell markers *CD4* and *CD8A* but not with B cell marker *CD19* (Fig. 8d–g). Consistent with the correlation with inflammatory cell markers, we also observed a negative correlation with cytokine expression—for example, *TNF*, *CCL2*, *CCL5* and *CXCL10*—as well as intercellular adhesion molecule 1 (*ICAM1*; Fig. 8h–l). Although the incidence of AAA is higher in males than in females, we did not observe any significant differences between those groups when comparing correlation of *AMOTL2* with inflammatory markers (Extended Data Fig. 10g–o).

## Discussion

The vascular endothelium plays an important role in the biomechanical response to hemodynamic forces. Understanding the pathways involved in this response is of importance to comprehend the pathogenesis of vascular disease. In this report, we show, to our knowledge for the first time, that the cellular junctions of arterial ECs are connected via AmotL2 and microfilaments to the nuclear lamina. Interference with this pathway impairs EC alignment in response to shear stress and abrogates nuclear positioning, resulting in inflammation and formation of AAAs.

We used an inducible mouse model to target AmotL2 in the EC lineage. In our previous publication^23^, we showed that Amotl2 silencing in ECs in utero resulted in impaired aortic expansion and death at embryonic day 10. Silencing of AmotL2 in adult mice, however, did not affect overall survival or have any obvious negative effects up to 6 months after AmotL2 depletion. The defect in adult mice was clearly more subtle as it was restricted to ECs exposed to arterial flow. We not only demonstrated that AmotL2 is required for cellular alignment in areas of shear stress but also provided insights into how VE-cadherin is mechanically coupled to the cytoskeleton and, thereby, controls cell shape. AmotL2 triggers the formation of radial actin filaments that mediate junctional tension between neighbouring cells. These radial actin filaments were detected in arterial but not, or at least at lower levels, in venous endothelium. AmotL2 is a scaffold protein and, as such, brings together protein complexes of different functions, such as Par3, MAGI-1b, Merlin, actin and VE-cadherin. Of interest is that, in HUVECs of the venous origin, AmotL2 is sufficient to induce radial actin filaments when the cells aligned under flow condition. Our data are consistent with the notion that VE-cadherin is part of a mechanosensory complex with VEGFR2 and PECAM1, as previously described^41^. The present data show that the VE-cadherin/AmotL2 protein complex is responsible for the actual cell shape modulation in arterial ECs. These most recent investigations, together with our previous findings, show that the VE-cadherin/AmotL2 complex mediates mechanical forces between ECs, suggesting that AmotL2 may not only relay mechanical forces from low wall shear stress, as has been associated with AAA rupture shear stress, but also transfer mechanical signals between cells.

Of particular interest is the observation that AmotL2 is required for nuclear shape and subcellular positioning. Ingber et al. showed early on that there is a direct linkage between the cytoskeleton and the cell nucleus, opening the possibility of a mechanical signalling pathway from the exterior to the nucleus^42^. Actin filaments are directly associated with the nuclear lamina by binding to Nesprin2, SUN1 and SUN2 and Lamin A that form the LINC complex. The actin filaments are anchored to the nuclear lamina via the LINC complex to form linear punctae called TAN lines (Fig. 8m). In this report, we present evidence for an as yet uncharacterized pathway that mechanically links junctional proteins to the nuclear LINC complex. We show that, concomitant with the loss of AmotL2 and radial actin filaments, nuclei lose their central position and translocate to a polarized position in the cell, downstream of the exerted flow direction. Arterial nuclei not only lose their subcellular positioning, but also the integrity of the nuclear lamina is perturbed. Measurement of forces exerted on the LINC proteins suggest that nuclear positioning is a result of the dynamic interactions of the cytoskeleton where nuclei are exposed to constant actomyosin forces^43^. Depletion of AmotL2 also had consequences for the integrity of the nuclear lamina. The nuclear lamina is an intermediate filament meshwork composed of two types of lamin proteins, the B type (Lamin B1 and B2) and the A type (Lamin A/C), and associated inner nuclear membrane proteins^44^. This network determines the mechanical properties and morphology of the nucleus^45^. In particular, Lamin A has been proposed to be responsive to mechanical cues from the extracellular matrix. Reduction of Lamin A in the nuclear membrane also results in a less rigid nuclear membrane.

The lack of alignment and the consequent irregular shapes of the arterial endothelium, concomitant with the loss of nuclear positioning, had direct consequences for the endothelial function. We showed that AmotL2-deficient cells acquire a pro-inflammatory phenotype with ensuing formation of areas of vascular inflammation characterized by the presence of Cd45^+^ monocytes. Quantitative PCR analysis revealed a sex-specific difference in the pro-inflammatory response. Although inflammation is generally considered a negative factor in the development of AAA, some studies have suggested that certain aspects of inflammation may have a protective effect, as seen with Cxcl10, which has been shown to have a protective role in AAA^46^. Although the reason for AmotL2 deficiency triggering deleterious or protective inflammatory responses remains unclear, the sex specificity of these responses may be related to estrogen levels, which have been shown to influence aortic disease^47^. Of clinical importance is the formation of AAA in the *amotl2*^*ec*−*/ec*−^ male mice. This is of interest as AAA has a relatively high prevalence in males 65–79 years of age, and the rupture of AAA is the cause of over 15,000 deaths per year in the United States alone and 175,000 globally^2,48–50^. Our data indicate a lower expression of AmotL2 in AAA from patients as compared to healthy aortas. Several mouse models of AAA have been established for developing therapies for AAA. However, so far, these models have failed to reliably predict results in clinical trials. The mouse model presented here is unique in the aspect that inactivation in aortic EC promotes inflammation and spontaneous aneurysm formation specifically in male mice.

We propose that lack of EC alignment due to AmotL2 deficiency activates pro-inflammatory markers such as Vcam1 and Icam1 on the EC surface. This promotes the extravasation of Cd45^+^ inflammatory cells. We speculate that the presence of inflammatory cells in the tunica media promotes the degradation of the extracellular matrix such as elastin and, thus, weakens the physical strength of the aortic wall. The weakened area of the artery then bulges out and poses an increased risk of blood vessel rupture and hemorrhage (as shown in the schematic in Fig. 8n).

At present, it is not clear whether there is a genetic association between AmotL2 and AAA. It is also possible that other mechanisms, such as epigenetic or environmental factors, may still play a role, and further research is needed to fully comprehend the relationship between AmotL2 and AAA. If lower levels of AmotL2 do indeed increase the risk of vascular inflammation, it would open up the potential to restore the AmotL2 mechanotransduction pathway as a therapeutic approach to enhance the arterial wall’s resilience to shear stress.

## Methods

This research complies with all relevant ethical regulations by the Regional Ethical Review Board in Stockholm, the Stockholm North Animal Experiment Ethics Board and the Swedish Board of Agriculture.

### Human material

Aortic samples from patients with AAA were obtained from surgeries performed at Karolinska Hospital in Stockholm, Sweden. Signed consent from patients with AAA was obtained for tissue collection. Control samples were taken from the abdominal aorta of beating-heart, solid organ transplant donors. Organ donors consented to the use of tissue for research purposes at the time of enlisting to the donation registry. Ethical permission was granted by the Regional Ethical Review Board in Stockholm. No participant compensation was granted.

RNA was extracted from both medial and adventitia layers of the aortic wall and subsequently sequenced on Human Transcriptome Array 2.0–Affymetrix (HTA 2.0) platform^51^.

### Mice and tamoxifen injections

The *amotl2*^*flox/flox*^ mice, carrying a loxP-flanked *amotl2* gene, were crossed to Cdh5(PAC)^CreERT2^ and ROSA26-EYFP double transgenic mice. To induce endothelial-specific *amotl2* gene inactivation, tamoxifen (100 µl, 20 mg ml^−1^) was administered by intraperitoneal injection for five continuous days in adult mice aged over 6 weeks. Analysis of mice samples was performed 2–6 weeks after injections. All the mice in this report had C57BL/6 background, and both females and males were included. The age of the mice used for different experiments is indicated in the respective figure legends. Ethical permits (N129/15, 12931-2020 and 22902-2021) were approved by the Stockholm North Animal Experiment Ethics Board, and all experiments were carried out in accordance with the guidelines of the Swedish Board of Agriculture.

### Local PPE model of aorta aneurysm

Tamoxifen injections (5 d) were administered to mice aged 9 weeks to induce endothelial-specific *amotl2* gene inactivation. At 12 weeks, mice were induced with 2–3% isoflurane anaesthesia in the chamber, placed on and fixed to a heating pad and then maintained with 1.5% isoflurane anaesthesia during surgery. The abdominal aorta from just below the left renal vein to the iliac bifurcation was identified and dissected peripherally from about 2 mm below the left renal nerve to the bifurcation. Topical local application of 5 μl of elastase from porcine pancreases (10.1 mg of protein per milliliter, 19 U mg^−1^ protein) was used to the exposed aortic adventitia for 5 min. Afterwards, the aortas were dried with a cotton swab and gently washed with warm 0.9% saline. The intestines were returned to the abdominal cavity, and the laparotomy was closed.

The CODA mouse tail-cuff system (Kent Scientific) was used for the measurement of hemodynamic parameters, including blood pressure and heart rate, once a week from week 9 to post-surgery day 28 (week 16). Mice had been trained in advance to adjust the tail-cuff system. Ultrasound (Vevo 2100) was performed for the visualization of vascular disease and to measure the aortic diameter under isoflurane anaesthesia at the day before surgery and on days 7, 14, 21 and 28 after surgery (weeks 13–16). At 16 weeks, the experiments reached their endpoint.

### Tissue preparation

The mice were euthanized using carbon dioxide. Their thoracic cavities were rapidly opened, and their hearts were exposed while still beating. (1) For the whole-mount staining of aortas, cold PBS was injected through a cannula for perfusion for 1 min and then changed to 4% paraformaldehyde (PFA) for another 1 min. Each aorta was dissected from root to aortic-common iliac bifurcation. This was followed by the careful removal of the connective tissues. After 1 h of extra fixation in 4% PFA, the entire aortas were opened longitudinally. For the aortic arches, the inner curvatures were cut along anteriorly using spring scissors, whereas the outer curvatures of the aortas were opened from the aortic root through the innominate, carotid and subclavian arteries until the aortic arch resembled a Y-shape split. The whole flattened-out aortas were pinned onto the wax molds and prepared for immunostaining. (2) For the paraffin section of aortas, an extra 24 h of fixation at 4 °C was applied after perfusion with 4% PFA, and then the samples stayed in 70% ethanol until paraffin embedding. (3) Aortas used for the cryosections were perfused with cold PBS for 1 min before dissection. The infrarenal dilated aortas were harvested, embedded in optimal cutting temperature (OCT) compound and frozen at −80 °C for subsequent sectioning and immunostaining. (4) For mRNA and protein isolation, the aortas were perfused with cold PBS for 2 min before careful dissection. Ascending thoracic and descending aortas were removed and frozen at −80 °C for subsequent extraction.

For the scRNA-seq experiment, after 1 min of PBS perfusion at room temperature the aortas were dissected. The lumen side was exposed and digested (1 mg ml^−1^ collagenase I, 1 mg ml^−1^ dispase and 150 μg ml^−1^ DNase-I) for 30 min at 37 °C. The suspension was passed through a 70-μm cell strainer for a single-cell solution. Cd45^−^ cells were negatively purified by Cd45 magnetic beads (15-min incubation). To further purify Cd31^+^Cd45^−^ ECs, Cd31 antibodies and eFluor 450 viability dye were applied for FACS according to the manufacturer’s protocol. The figure that exemplifies the gating strategy is provided as Supplementary Fig. 1. A few Cd31^−^Cd45^−^ cells were sorted as controls. Single cells were sorted into 384-well plates containing a lysis buffer compatible with Smart-seq2. The plates were centrifuged, snap frozen on dry ice and stored at −80 °C.

To isolate the urinary bladder, the mice were handled especially gently before tissue harvest, which prevented urine leakage. Full bladders were immersed in fixative for 2 h and pinned on a wax mold in an open flower shape for whole-mount staining.

Mouse eyeballs were dissected out intact. After a 2-h fixation in 4% PFA, the retinas were dissected out and prepared for immunofluorescence staining for vasculature analysis.

### Cell culture

Murine ECs (MS1, purchased from the American Type Culture Collection, CRL-2279) were cultured in RPMI 1640 medium supplemented with 10% FBS and 1% penicillin–streptomycin. BAE cells (Sigma-Aldrich, B304-05) were cultured in a bovine endothelial cell growth medium. HUVECs from ScienCell (8000) were cultured in endothelial cell medium. HAoECs from PromoCell (C-12271) were cultured with Endothelial Cell Growth Medium MV. The batch of HAoECs used for this study came from a 55-year-old male donor with a Caucasian background. The cell media mentioned above are listed in Supplementary Table 11.

### Lentiviral-induced knockdown

For knockdown studies, HAoECs were transfected with customized AmotL2 short hairpin RNA (shRNA) lentiviral particles or scrambled control shRNA lentivirus in complete EC culture medium with polybrene (5 μg ml^−1^, VectorBuilder). The lentivirus-containing medium was removed after overnight incubation, and fresh medium was added. Further analyses of confluent cells were performed at ≥72 h after transfection.

### Flow experiments

#### ibidi flow system

Flow chamber slides (ibidi μ‐Slides VI 0.4 ibidi‐treated) with a volume of 30 μl per parallel channel were coated with fibronectin. HAoECs/HUVECs were grown on the slides for 24 h until 30–50% confluency, which was followed by lentivirus transfection. Then, 48 h later, cells were subjected to 14 dynes per cm^2^ laminar flow using the ibidi pump system (with pump control software) or kept in the same incubator statically for 48 h. Cells were then harvested and processed for further analysis.

#### Orbital shaker

A Rotamax 120 (Heidolph) generating circular motion with the maximum speed of 300 r.p.m. was used. HAoECs and HUVECs on a 15-cm culture dish with 16 ml of medium were placed on the shaker for 96 h in the incubator before they were harvested.

### Rho activity

Active Rho Detection Kit was purchased to measure the activation of Rho GTPase in the cell. A GST-Rhotekin-RBD fusion protein was used to bind the active, GTP-bound form of Rho, which could then be pulled down by glutathione resin co-IP. Cells grown at the periphery (from 6 cm to the edge of a 15-cm dish) were harvested and employed according to the manufacturer’s protocol. The activation levels of Rho were then tested by western blot using a Rho rabbit antibody.

### IF staining and the PLA

IF staining was performed on cells at the monolayer. In brief, cells were fixed with 4% PFA for 10 min and permeabilized with 0.1% Triton X-100 for 1 min. After blocking in 5% horse serum in PBS for 1 h, primary antibodies were diluted in the blocking solution and incubated overnight at 4 °C. Secondary antibodies were subjected afterwards for 1 h in room temperature before mounting with medium containing DAPI. Three times of 5-min washing were performed between each step.

To stain open aorta pinned on wax, endothelium exposed on the top layer was carefully treated using the same protocol as for cell staining, with the exception that each aorta was permeabilized for 20 min with 0.1% Triton X-100 in PBS.

Retinas and bladders were blocked and permeabilized in 1% BSA and 0.3% Triton X-100 in PBS overnight. Pblec buffer (1.0% Triton X-100 plus 0.1 M MgCl_2_, 0.1 M CaCl_2_, 0.01 M MnCl_2_ in PBS) was used to wash and incubate one or more primary antibodies. Then, fluorophore-conjugated antibodies were added to the blocking buffer, followed by five 20-min washes with the blocking buffer at 1:1 dilution in PBS. Finally, the cells and whole tissue were mounted using FluoroShield with DAPI.

PLA was performed using the NaveniFlex MR Kit (Navinci Diagnostics) after 10-min fixation and 10-min permeabilization on whole-mounted aortic tissue. After blocking (37 °C, 1 h), the primary antibody (4 °C overnight) was applied. The next procedures were performed as instructed by the manufacturer’s protocol. Phalloidin was added to visualize actin filaments (room temperature, 1 h), and then three more washings and mounting were performed.

A Zeiss LSM 700 confocal microscope was used to acquire digital images. Airyscan-resolution microscopy (Zeiss LSM 980 Airyscan) was applied to capture high-resolution images. Images were analysed using ImageJ.

### Western blot

Cells were scraped directly from the culture dish in lysis buffer (50 mM HEPES buffer, 150 mM NaCl, 1.5 mM MgCl_2_, 1 mM EGTA, 10% glycerol, 1% Triton X-100) with 1× protease inhibitor and optionally with Phosphatase Inhibitor Cocktail 1. Lysates were prepared with an SDS sample buffer containing 10% sample reducing agent, separated in a polyacrylamide gel with 4–12% gradient and transferred to a nitrocellulose membrane. The membrane was blocked in 5% non-fat milk PBS with 0.1% Tween 20 and sequentially incubated with the primary antibody at 4 °C overnight. Horseradish peroxidase (HRP)-conjugated secondary antibody was added so that labelled proteins could be detected by Western Lightning Plus-ECL.

### Co-IP analysis

Mouse lung tissues were cut into small pieces before being transferred into the lysis buffer (50 mM HEPES buffer, 150 mM NaCl, 1.5 mM MgCl_2_, 1 mM EGTA, 10% glycerol, 1% Triton X-100). Tissue homogenizer was gently applied for better protein extraction. Cell and tissue lysates were incubated with Protein G Sepharose beads for 1.5 h at 4 °C as a pre-cleaning. Then, 2 µg of Amotl2 or control IgG were added to the lysates and incubated overnight at 4 °C. The next morning, the immunocomplexes were pulled down by Protein G beads for 2 h at 4 °C, followed by five washes with lysis buffer. The final protein samples were fractionated by polyacrylamide gel, and the fractions were probed in western blot to evaluate immunoprecipitated proteins.

### Proximity-dependent BioID

Proximity-dependent BioID plasmids (Mammalian Gene Expression Lentiviral Vector) were constructed by combining cDNA fragments encoding human p100-AmotL2 (accession no: NM_001278683) or p60-AmotL2 with N-terminus of *E. coli* biotin ligase (BirA). p60-AmotL2, in contrast to p100-AmotL2, contains the del760A alteration (370 amino acids missing in the N-terminal). An empty vector with the same backbone was used as negative control. All constructs were verified by restriction enzyme digestion. BioID constructs and the empty vector were packaged into lentivirus using Lipofectamine 3000 Transfection Reagent. MS1 cells were used to establish stable transfected cell lines via lentivirus transfection with the selection of 0.5 mg ml^−1^ geneticin. Stable transfected cells were cultured in RPMI 1640 medium without biotin, supplemented with 10% FBS and 0.5 mg ml^−1^ geneticin. For the BioID experiment, MS1 cells were treated with 50 mM biotin for 16 h, followed by harvesting in a lysis buffer consisting of 50 mM Tris-HCl pH 7.4, 8 M urea, 1 mM DTT and protease inhibitors. Then, 1% Triton X-100 was added to lysates before sonication. Biotinylated proteins were purified with streptavidin beads overnight at 4 °C. After five washes with 8 M urea in 50 mM Tris-HCl (pH 7.4) and one wash with 50 mM Tris-HCl (pH 7.4), the beads were resuspended in PBS and kept ready for further protein analysis. Three independent experiments were performed (*n* = 3 in all groups), including ‘p100-AmotL2 ± biotin’, ‘p60-AmotL2 ± biotin’ and ‘empty vector ± biotin’.

Protein identification criteria for BirA p100-AmotL2 BioID construct—that is, the ‘p100-AmotL2 + biotin’ group—were as follows: (1) a positive value in all triplicates and (2) a higher average value than the ones in the ‘empty vector ± biotin’, ‘p60-AmotL2 ± biotin’ and ‘p100-AmotL2 + biotin’ groups. In addition, (3) common contamination proteins were excluded (Krt1, Krt5, Rpl6, Rpl10a and Hmga2).

### MS analysis

IP and BioID samples were prepared by on-bead reduction, alkylation and digestion (trypsin, sequencing grade modified, Pierce) followed by SP3 peptide cleanup^52^. Each sample was separated using a Thermo Fisher Scientific Dionex nano LC system in a 3-h 5–40% ACN gradient coupled to a Thermo Fisher Scientific HF Q Exactive (see below for detailed liquid chromatography with tandem mass spectrometry (LC–MS/MS) parameters). Proteome Discoverer version 1.4 software, including Sequest-Percolator for improved identification, was used to search the *Mus musculus* or *Canis familiaris* UniProt database for protein identification, limited to a false discovery rate (FDR) of 1%.

For mouse ascending and descending aortic proteomics analysis, samples were homogenized using cryoPREP dry tissue pulveriser from Covaris, lysed by Qiagen AllPrep Kit RLT buffer. The Qiagen AllPrep Kit was used for RNA and DNA isolation, and the protein fraction of each sample was prepared for MS analysis using a modified version of the SP3 protein cleanup and digestion protocol^52^. In brief, each sample was alkylated with 4 mM chloroacetamide, and Sera-Mag SP3 bead mix (20 µl) was transferred into the protein sample together with 100% acetonitrile to a final concentration of 70%. The mix was incubated under rotation at room temperature for 20 min. The mixture was placed on the magnetic rack, and the supernatant was discarded, followed by two washes with 70% ethanol and one with 100% acetonitrile. The beads–protein complex was reconstituted in 100 µl of trypsin buffer (50 mM HEPES pH 7.6 and 0.8 µg of trypsin) and incubated overnight at 37 °C. Peptides were labelled with tandem mass tag (TMT) 16plex reagent according to the manufacturer’s protocol (Thermo Fisher Scientific) and separated by immobilized pH gradient isoelectric focusing (IPG-IEF) on 3–10 strips^53^.

Online LC–MS was performed using a Dionex UltiMate 3000 RSLCnano system coupled to a Q Exactive HF mass spectrometer (Thermo Fisher Scientific). IP samples were trapped on a C18 guard-desalting column (Acclaim PepMap 100, 75 μm × 2 cm, nanoViper, C18, 5 µm, 100 Å) and separated on a 50-cm-long C18 column (EASY-spray PepMap RSLC, C18, 2 μm, 100 Å, 75 μm × 50 cm). The nano capillary solvent A was 95% water, 5% DMSO and 0.1% formic acid; solvent B was 5% water, 5% DMSO, 90% acetonitrile and 0.1% formic acid. At a constant flow of 0.25 μl min^−1^, the curved gradient went from 2% B up to 40% B in 240 min, followed by a steep increase to 100% B in 5 min.

FTMS master scans with 70,000 resolution (and mass range 300–1,700 *m*/*z*) were followed by data-dependent MS/MS (35,000 resolution) on the top five ions using higher-energy collision dissociation (HCD) at 30–40% normalized collision energy. Precursors were isolated with a 2-*m*/*z* window. Automatic gain control (AGC) targets were 1 × 10^6^ for MS1 and 1 × 10^5^ for MS2. Maximum injection times were 100 ms for MS1 and 150–200 ms for MS2. The entire duty cycle lasted ~2.5 s. Dynamic exclusion was used with 60-s duration. Precursors with unassigned charge state or charge state 1 were excluded, and an underfill ratio of 1% was used.

Extracted peptide fractions from the IPG-IEF were separated using an online 3000 RSLCnano system coupled to a Thermo Fisher Scientific Q Exactive HF mass spectrometer. MSGF+ and Percolator in the Nextflow platform were used to match MS spectra to the Ensembl_105 *Mus musculus* protein database. The quantification of TMT 16plex reporter ions was done using OpenMS project’s IsobaricAnalyzer (version 2.0)^54^. Peptide–spectrum matches (PSMs) found at 1% FDR were used to infer gene identities. Protein quantification by TMT 16plex reporter ions was calculated using TMT PSM ratios to the entire sample set (all 16 TMT channels) and normalized to the sample median. The median PSM TMT reporter ratio from peptides unique to a gene symbol was used for quantification. Protein FDRs were calculated using the picked FDR method using gene symbols as protein groups and limited to 1%^55^.

### qRT–PCR

To extract RNA, dissected aortas from *amotl2*^*ec+/ec+*^ (*n* = 3) and *amotl2*^*ec*−*/ec*−^ mice (*n* = 3) were immersed in TRIzol and homogenized by TissueLyser (Qiagen). Chloroform addition allowed the homogenate to separate into the lower organic phase and the upper clear aqueous phase (containing RNA). Cells cultured in vitro were scraped directly from the culture dish in RLT buffer.

Total RNA purification was carried out using RNeasy Plus Mini Kit (Qiagen). cDNA synthesis was performed using the High-Capacity cDNA Reverse Transcription Kit. Quantitative real-time PCR was performed on a 7900HT Fast Real-Time PCR system using TaqMan Assay-on-Demand (in vivo samples) and QuantStudio 7 Flex Real-Time PCR System using SYBR Green PCR Master Mix (in vitro samples). The results were calculated as 2 − ΔCT obtained by comparing the cycle threshold (CT) for the genes of interest with those obtained for the housekeeping gene *Hprt*/*HPRT* (used for all qPCR analyses).

### RNA-seq

The total RNA purified from aortic tissues and HAoECs were sent for RNA-seq analysis (Novogene). Libraries were prepared from 4–5 µg of total RNA. Poly(A) RNA was purified using the Dynabeads mRNA Purification Kit and fragmented using Fragmentation Reagent (Ambion). First-strand cDNA was synthesized from poly(A) RNA using the SuperScript III Reverse Transcriptase Kit with random primers (Life Technologies). Second-strand cDNA synthesis was performed using Second Strand Synthesis buffer, DNA Pol I and RNase H (Life Technologies). cDNA libraries were prepared for sequencing using the mRNA TruSeq protocol (Illumina).

The genes with significantly differential expression were input into Enrichr (online Ma’ayan Laboratory, Computational Systems Biology) for GO term analysis and KEGG pathway analysis.

### scRNA-seq

Single-cell mRNA libraries were generated with Smart-seq2 (refs. ^56,57^). In brief, primer annealing was followed by RT and cDNA amplification. Clean-up of PCR products, tagmentation of cDNA and amplification of the final library were performed using custom barcoded primers. Libraries were pooled and cleaned up with SPRI beads and then sequenced on an Illumina NextSeq 550.

RNA reads were aligned to GRCm38 with added External RNA Controls Consortium (ERCC) spike-ins and the reporter gene EYFP, using STAR 2.7.7a^58^. Duplicate reads were removed using Picard 2.22.0 (ref. ^59^), and read counts were summarized using HTSeq 0.9.1 (ref. ^60^). Cutoffs of 20,000 counts and 500 features were used. Cells were then processed with Seurat 4.1.1 (ref. ^61^). The data were normalized and scaled, and linear dimensionality reduction was performed using the first 30 dimensions. Seuratʼs FindNeighbors function, with 30 dimensions, was used to construct a *k*-nearest neighbour graph, and FindClusters, with the resolution set to 0.5, was used to cluster the cells. To confirm cell types, *Cdh5* was used to identify ECs. FindAllMarkers from Seurat was used to find marker genes for each cluster with the thresholds: adjusted *P* < 0.05 and log_2_ fold change > 1. Identified marker genes were subjected to KEGG pathway analysis (Enrichr). GeneScore analysis was performed with AddModuleScore from Seurat using the default parameters. GO terms were derived from http://geneontology.org/.

### Materials

Purchase information of reagents and kits, lentivirus-based shRNA constructs, TaqMan probes and SYBR Green qPCR primers used in this study is provided in Supplementary Tables 11–14, respectively.

### Statistics and reproducibility

All statistical figures and analyses were made using GraphPad Prism software, except for the gene correlation graphs, which were generated using R (https://www.r-project.org/index.html). The statistical analysis of in vivo results was based on at least three animals per group. Comparisons between two groups with similar variances were made using the standard unpaired two-tailed Student *t*-test, whereas comparisons between multiple groups were made using the Kruskal–Wallis test. A Wilcoxon test was used to compare gene scores between clusters in the scRNA-seq experiments. Statistical analysis of scRNA data was performed using RStudio 2022.02.2+485. The correlation between two genes was analysed using the Pearson correlation, and Pearson correlation coefficient was referred to as *r*. *P* value and *r* (microarray analysis in patients with AAA) was calculated using R version 4.1.1. A value of *P* < 0.05 was considered statistically significant (NS, not significant, ^*^P < 0.05, ^**^P < 0.01 and ^***^P < 0.001).

All box plots (data points more than or equal to 10) presented in this paper are the min–max box plot, which shows the five-number summary of a dataset, including the minimum (smallest whiskers), the first quartile, the median (centre line), the third quartile and the maximum (largest whiskers). The bar graphs with individual data points were employed when the number of samples was less than 10.

All the results from western blot and co-IP experiments in this study were observed at least in three independent experiments.

### Reporting summary

Further information on research design is available in the Nature Portfolio Reporting Summary linked to this article.

### Supplementary information

Supplementary information Supplementary Fig. 1 and Supplementary Tables 11–14

Reporting Summary

Supplementary Table 1 MS analysis of AmotL2 IP in MS1 cells (original data). Related to Fig. 4a.

Supplementary Table 2 MS analysis of AmotL2 IP in BAE cells (original data). Related to Extended Data Fig. 4a.

Supplementary Table 3 MS analysis of AmotL2 BioID in MS1 cells. Related to Fig. 4c.

Supplementary Table 4 Differentially expressed genes analysed (AmotL2 KO versus WT aortas) with enriched GO. Related to Fig. 5b.

Supplementary Table 5 Differentially expressed gene list and KEGG pathway analysis between cluster 5 versus cluster 4 in scRNA-seq data. Related to Fig. 6e.

Supplementary Table 6 Differentially expressed gene list and KEGG pathway analysis between cluster 5 versus cluster 3 in scRNA-seq data. Related to Extended Data Fig. 7g.

Supplementary Table 7 The upregulated gene list and KEGG pathway analysis between shAmotL2 versus shControl under flow condition in RNA-seq data. Related to Fig. 6f.

Supplementary Table 8 Downregulated gene list and KEGG pathway analysis between flow versus static condition in shControl and shAmotL2 transfected HAoECs in RNA-seq data. Related to Extended Data Fig. 8b.

Supplementary Table 9 Differentially expressed genes and KEGG pathway analysis between DA versus ATA in RNA-seq data (590 genes). Related to Extended Data Fig. 9c.

Supplementary Table 10 Differentially expressed genes and GO cellular component analysis between DA versus ATA in MS data. Related to Extended Data Fig. 9f.

### Source data

Source Data Fig. 1 Statistical Source Data

Source Data Fig. 2 Statistical Source Data

Source Data Fig. 3 Statistical Source Data

Source Data Fig. 4 Statistical Source Data

Source Data Fig. 4 Unprocessed western blots

Source Data Fig. 5 Statistical Source Data

Source Data Fig. 6 Statistical Source Data

Source Data Fig. 7 Statistical Source Data

Source Data Fig. 8 Statistical Source Data

Source Data Extended Data Fig./Table 1 Statistical Source Data

Source Data Extended Data Fig./Table 2 Statistical Source Data

Source Data Extended Data Fig./Table 3 Statistical Source Data

Source Data Extended Data Fig./Table 3 Unprocessed western blots

Source Data Extended Data Fig./Table 4 Statistical Source Data

Source Data Extended Data Fig./Table 4 Unprocessed western blots

Source Data Extended Data Fig./Table 5 Unprocessed western blots

Source Data Extended Data Fig./Table 6 Statistical Source Data

Source Data Extended Data Fig./Table 7 Statistical Source Data

Source Data Extended Data Fig./Table 8 Statistical Source Data

Source Data Extended Data Fig./Table 9 Statistical Source Data

Source Data Extended Data Fig./Table 10 Statistical Source Data

## Data Availability

The authors declare that all sequencing data supporting the findings of this study have been deposited in the National Center for Biotechnology Information Gene Expression Omnibus (GEO) and Sequence Read Archive (SRA). All original RNA sequencing data are available in the SRA with BioProject accession numbers PRJNA916890, PRJNA914693 and PRJNA914653. scRNA-seq data have been deposited in the GEO with series accession number GSE222159. The human AAA expression analysis was presented previously by Lindquist-Liljeqvist et al.^51^ with the original microarray data deposited in the GEO (accession number GSE232911). The mass spectrometry proteomics data have been deposited to the ProteomeXchange Consortium via the PRIDE^62^ partner repository with the dataset identifier PXD039256 and PXD042661. All other data supporting the findings in this study are included in the main article and associated Source Data files.

## Code availability

Polar chart for analysing cellular and nuclear orientation was coded by A. Gustafsson using Python. Scripts to reproduce the analysis presented in this study have been deposited on GitHub (https://github.com/tokko/PolarCharts).
