# Supplementary material for: The VE-cadherin/AmotL2 mechanosensory pathway suppresses aortic inflammation and the formation of abdominal aortic aneurysms
Source: Nat Cardiovasc Res. 2023 Jun 29;2(7):629–44. doi: 10.1038/s44161-023-00298-8 (PMC11358041; doi:10.1038/s44161-023-00298-8)
Supplement: Supplementary file 1 — Supplementary Fig. 1 and Supplementary Tables 11–14 [file 44161_2023_298_MOESM1_ESM.pdf]

nature cardiovascular research

Article https://doi.org/10.1038/s44161-023-00298-8

# The VE-cadherin/AmotL2 mechanosensory pathway suppresses aortic inflammation and the formation of abdominal aortic aneurysms

In the format provided by the authors and unedited

# Supplementary Figure 1. FACS sorting-gating strategy.

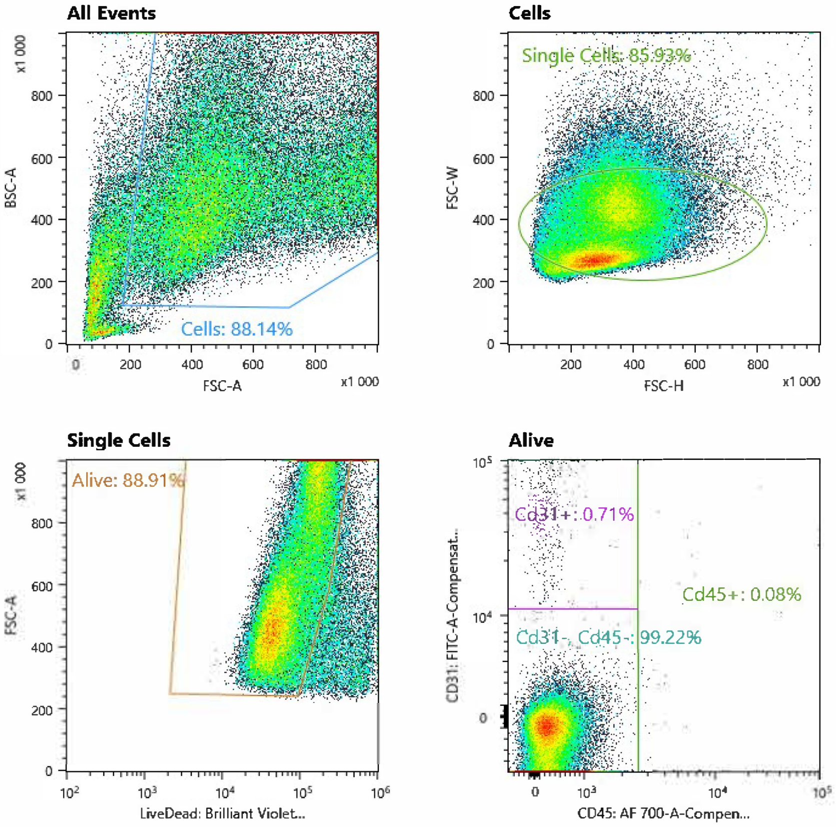


Gates and Statistics

| Name | Events | %Parent | %Total |
|---|---|---|---|
| All Events | 116,155 | 0.00% | 100.00% |
| Cells | 102,380 | 88.14% | 88.14% |
| Single Cells | 87,980 | 85.93% | 75.74% |
| Alive | 78,223 | 88.91% | 67.34% |
| Cd31-, Cd45- | 77,612 | 99.22% | 66.82% |
| Cd31+ | 554 | 0.71% | 0.48% |
| Cd45+ | 60 | 0.08% | 0.05% |

## Supplementary Table 11. Reagents/Kits

| **Reagents** | | |
|---|---|---|
| **Reagent** | **Company** | **Catalog number** |
| *In vivo* | | |
| Elastase from porcine pancreas | Sigma | E7885 |
| Tamoxifen | Sigma | T5648 |
| *In vitro* | | |
| Paraformaldehyde solution 4% in PBS | ChemCruz | sc-281692 |
| Fluoroshield with DAPI | Sigma | F6057 |
| Protein G Sepharose 4 fast flow | GE Healthcare | 17-0618-01 |
| Streptavidin Sepharose High Performance Beads | GE Healthcare | 17511301 |
| IgG from rabbit serum | Sigma | I8140 |
| IgG from mouse serum | Sigma | I8765 |
| Polybrene (Hexadimethrine Bromide) | VectorBuilder | provided with lentivirus |
| Polybrene (Hexadimethrine Bromide) | Sigma | H9268-5G |
| Collagenase, Type I, powder | Gibco | 10114532 |
| Dispase II, powder | Gibco | 11510536 |
| DNase I | Sigma | 10104159001 |
| CD45 MicroBeads, mouse | Miltenyi Biotec | 130-052-301 |
| Geneticin® 50mg/mL | Gibco | 10131-035 |
| Biotin | Sigma | B4501-1G |
| Urea | Sigma | U5378-1KG |
| Western blotting | | |
| Western Lightning Plus-ECL | PerkinElmer | 203-170071 |
| Phosphatase Inhibitor Cocktail 1 | Sigma | P2850-1ML |
| cOmplete™, Mini, EDTA-free Protease Inhibitor Cocktail | Roche | 4693159001 |
| NuPAGE™ LDS Sample Buffer (4X) | Invitrogen | NP0007 |
| NuPAGE™ Sample Reducing Agent (10X) | Invitrogen | NP0009 |
| NuPAGE™ 4 to 12%, Bis-Tris, 1.0–1.5 mm, Mini Protein Gels | Invitrogen | NP0335BOX |
| Cytiva Amersham™ Protran™ NC Nitrocellulose Membranes: Rolls | Fisher Scientific | 15249794 |

| Cell culture | | |
|---|---|---|
| **Reagent** | **Company** | **Catalog number** |
| FBS | Gibco | 10270-106 |
| Penicillin-Streptomycin (10,000 U/mL) | Gibco | 15140-122 |
| RPMI 1640 Medium | Gibco | 21875-034 |
| R9002-01 RPMI 1640 Medium Modified w/L-Glutamine w/o Phenol, Red Biotin (Powder) | Usbiological | R9002-01 |
| Bovine Endothelial Cell Growth Medium | Sigma | B211-500 |
| Endothelial Cell Growth Medium MV | PromoCell | C-22020 |
| Endothelial cell Medium | ScienCell | #1001 |
| Fibronectin human plasma | Sigma | F0895-5MG |
| | | |
| **Kits** | | |
| **kits** | **Company** | **Catalog number** |
| Active Rho Detection Kit | Cellsignal | #8820 |
| Nitric Oxide Assay Kit | Invitrogen | EMSNO |
| NaveniFlex MR kit | Navinci Diagnostics AB | N/A |
| Rneasy® Plus Mini Kit | QIAGEN | 74134 |
| High Capacity RNA-to-cDNA Kit | Applied Biosystems | 4387406 |
| Lipofectamine 3000 Transfection Reagent | Invitrogen | L3000015 |

## Supplementary Table 12. Lentivirus-based shRNA constructs

| Name | Company | Sequence if it's customized product |
|---|---|---|
| Non-Targeting (scrambled) shRNA Control | Sigma | MISSION® pLKO.1-puro Non-Target shRNA Control Plasmid DNA (catalog number: SHC016) |
| human AmotL2 shRNA virus | Sigma | 5′-GCGAGAGAAGGAGGAGCAGATC-3′ |
| Empty vector plasmid (BioID construct) | VectorBuilder | VB190314-1055uyu pLV[Exp]-Neo-CMV >Stuffer300 |
| Human p100-AmotL2 BirA-tagged plasmid (BioID construct) | VectorBuilder | pLV[Exp]-Neo-CMV>{BirA(R118G)}:hAMOTL2[NM_001278683.1 |
| Human p60-AmotL2 BirA-tagged plasmid (BioID construct) | VectorBuilder | pLV[Exp]-Neo-CMV>{BirA(R118G)}:{P60} |

## Supplementary Table 13. TaqMan probes

| | |
|---|---|
| **Company** | Thermo-Fisher |
| **Species** | Mouse |
| **Gene** | **Taqman reference** |
| TNF | Mm00443258_m1 |
| CD68 | Mm03047343_m1 |
| Cxcl10 | Mm00445235_m1 |
| Icam1 | Mm00516023_m1 |
| Vcam1 | Mm01320970_m1 |
| Cd8a | Mm01188922_m1 |
| Cd4 | Mm01185100_m1 |
| Ccl2 | Mm00441242_m1 |
| Ccl5 | Mm01302427_m1 |
| Cd19 | Mm00515420_m1 |
| IL6 | Mm00446190_m1 |
| HPRT | Mm03024075_m1 |

## Supplementary Table 14. SYBR green qPCR primers

| primer name | Sequence |
|---|---|
| ALCAM human Forward | 5'-TCCAGAACACGATGAGGCAGAC-3' |
| ALCAM human Reverse | 5'-GTAGACGACACCAGCAACAAGG-3' |
| IL6R human No.1 Forward | 5'-GACTGTGCACTTGCTGGTGGAT-3' |
| IL6R human No.1 Reverse | 5'-ACTTCCTCACCAAGAGCACAGC-3' |
| ITGB8 human No.1 Forward | 5'-CTGTTTGCAGTGGTCGAGGAGT-3' |
| ITGB8 human No.1 Reverse | 5'-TGCCTGCTTCACACTCTCCATG-3' |
| BMP4 human No.1 Forward | 5'-CTGGTCTTGAGTATCCTGAGCG-3' |
| BMP4 human No.1 Reverse | 5'-TCACCTCGTTCTCAGGGATGCT-3' |
| IL1RAP human No.1 Forward | 5'-CTGAGGATCTCAAGCGCAGCTA-3' |
| IL1RAP human No.1 Reverse | 5'-AGCAGGACTGTGGCTCCAAAAC-3' |
| GDF7 human No.1 Forward | 5'-GCAGCCGCTGTCTCCGCCTC-3' |
| GDF7 human No.1 Reverse | 5'-TCTGCGTCGTTAAGGCTGGACA-3' |
| NEGR1 human Forward | 5'-ACCAATGCGAGCCTGCCTCTTA-3' |
| NEGR1 human Reverse | 5'-GCTGGTGAAAGAGGACAGTGTC-3' |
| NECTIN3 human Forward | 5'-ATTCCCGCTTGGAAATGCCCAG-3' |
| NECTIN3 human Reverse | 5'-GCTGCTACTGTTTCATTTCCTCC-3' |
| CLDN1 human Forward | 5'-GTCTTTGACTCCTTGCTGAATCTG-3' |
| CLDN1  human Reverse | 5'-CACCTCATCGTCTTCCAAGCAC-3' |
| SDC2 human Forward | 5'-GCTCCAAAAGTGGAAACCACGAC-3' |
| SDC2 human Reverse | 5'-ATCCTCTTCGGCTGGGTCCATT-3' |
| CDH2 human Forward | 5'-CCTCCAGAGTTTACTGCCATGAC-3' |
| CDH2 human Reverse | 5'-GTAGGATCTCCGCCACTGATTC-3' |
| IL6 human Forward | 5'-AGACAGCCACTCACCTCTTCAG-3' |
| IL6 human Reverse | 5'-TTCTGCCAGTGCCTCTTTGCTG-3' |
| HPRT human Forward | 5'-CATTATGCTGAGGATTTGGAAAGG-3' |
| HPRT human Reverse | 5'-CTTGAGCACACAGAGGGCTACA-3' |
