# Supplementary material for: The VE-cadherin/AmotL2 mechanosensory pathway suppresses aortic inflammation and the formation of abdominal aortic aneurysms
Source: Nat Cardiovasc Res. 2023 Jun 29;2(7):629–44. doi: 10.1038/s44161-023-00298-8 (PMC11358041; doi:10.1038/s44161-023-00298-8)
Supplement: Supplementary file 2 — Reporting Summary [file 44161_2023_298_MOESM2_ESM.pdf]

nature portfolio

Corresponding author(s): Lars Holmgren

Last updated by author(s): May 22, 2023

Reporting Summary

Nature Portfolio wishes to improve the reproducibility of the work that we publish. This form provides structure for consistency and transparency in reporting. For further information on Nature Portfolio policies, see our Editorial Policies and the Editorial Policy Checklist.

Statistics

For all statistical analyses, confirm that the following items are present in the figure legend, table legend, main text, or Methods section.

n/a	Confirmed	
☐	☒	The exact sample size (*n*) for each experimental group/condition, given as a discrete number and unit of measurement
☐	☒	A statement on whether measurements were taken from distinct samples or whether the same sample was measured repeatedly
☐	☒	The statistical test(s) used AND whether they are one- or two-sided<br>*Only common tests should be described solely by name; describe more complex techniques in the Methods section.*
☒	☐	A description of all covariates tested
☐	☒	A description of any assumptions or corrections, such as tests of normality and adjustment for multiple comparisons
☐	☒	A full description of the statistical parameters including central tendency (e.g. means) or other basic estimates (e.g. regression coefficient) AND variation (e.g. standard deviation) or associated estimates of uncertainty (e.g. confidence intervals)
☐	☒	For null hypothesis testing, the test statistic (e.g. *F*, *t*, *r*) with confidence intervals, effect sizes, degrees of freedom and *P* value noted<br>*Give P values as exact values whenever suitable.*
☒	☐	For Bayesian analysis, information on the choice of priors and Markov chain Monte Carlo settings
☒	☐	For hierarchical and complex designs, identification of the appropriate level for tests and full reporting of outcomes
☐	☒	Estimates of effect sizes (e.g. Cohen's *d*, Pearson's *r*), indicating how they were calculated

Our web collection on statistics for biologists contains articles on many of the points above.

Software and code

Policy information about availability of computer code

Data collection	Flow cytometry was performed on SH800s, and sequencing was done using the Illumina nextseq 550 platform.<br>TaqMan qPCR was run by 7900HT Fast Real-Time PCR system and SYBR based qPCR by QuantStudio™ 7 Flex Real-Time PCR System.
Data analysis	All the box, bar and dot plots were made in GraphPad Prism 9 software (San Diego, USA).<br>Confocal Images were analyzed in ImageJ 1.52k (NIH)<br>GO term analyses and KEGG pathway analyses were either analyzed by Enrichr (online Ma'ayan Laboratory, Computational systems biology) or Nonogene (China).<br>Flow cytometry was analyzed with Sony Cell sorter Softwer 2.1.3. In scRNA-seq, RNA reads were analyzed with added ERCC-spike ins using STAR 2.7.7a58. Duplicate reads were removed using Picard 2.22.059 and read counts were summarized using HTSeq 0.9.160. Cells were then processed with Seurat 4.1.1, using Rstudio 2022.02.2+485. Statistical analysis of single cell data was performed using Rstudio 2022.02.2+485.<br>R version 4.1.1 was used for data analyses (human AAA mRNA expression)<br>Polar chart for analyzing cellular and nuclear orientation was coded using Pathon. The codes developed by Andreas Gustafsson were deposited in https://github.com/tokko/PolarCharts

For manuscripts utilizing custom algorithms or software that are central to the research but not yet described in published literature, software must be made available to editors and reviewers. We strongly encourage code deposition in a community repository (e.g. GitHub). See the Nature Portfolio guidelines for submitting code & software for further information.

Data

Policy information about availability of data

All manuscripts must include a data availability statement. This statement should provide the following information, where applicable:

- Accession codes, unique identifiers, or web links for publicly available datasets
- A description of any restrictions on data availability
- For clinical datasets or third party data, please ensure that the statement adheres to our policy

All original RNA-seq data (1, mouse descending aorta WT vs AmotL2 KO mice. BioProject accession: PRJNA916890. 2, Ibidi flow on Ctrl vs AmotL2 KD HAoECs. BioProject accession: PRJNA914693. 3, comparison between descending aorta and ascending thoracic aorta. BioProject accession: PRJNA914653) and scRNA-seq data (GEO Accession: GSE222159) have been deposited in NCBI's Gene Expression Omnibus (GEO). In scRNA-seq, RNA reads were aligned to GRCm38.
The human AAA expression analysis has been presented previously by Lindquist-Liljeqvist (Arterioscler Thromb Vasc Biol. 2020;40:2700–2713) with the original Microarray data deposited in GEO (accession number GSE232911).
The proteomics data (1. AmotL2 co-IP Mass Spec in MS1 cells. 2. AmotL2 co-IP Mass Spec in BAE cells. 3. p100 AmotL2 BioID analysis and 4. Protein profiling comparison between descending aorta and ascending thoracic aorta) have been deposited to PRIDE. Project accession: PXD042661(1,2, and 3) and PXD039256 (4).

Human research participants

Policy information about studies involving human research participants and Sex and Gender in Research.

Reporting on sex and gender	A total of 35 (35% women) AAA patients and 13 Donor controls (46% women). Gene expression analyses on both sexes are presented in Extended Data Fig. 10
Population characteristics	A total of 35 AAA patients and 13 Donor controls:<br>Age (y) (median): AAA: 70 (interquartile range: 66-76); Donor controls: 53 (interquartile range: 44-68)<br>Female: AAA: 9 (35%); Donor controls: 6 (46%)<br>Smoking: AAA: 12 (34%); Donor controls: 3 (23%)<br>Family history of AA: AAA: 3 (9%); Donor controls: Not known<br>Hypertension: AAA: 22 (63%); Donor controls: Not known<br>Stroke: AAA: 3 (9%); Donor controls: Not known<br>Previous Myocardial infarction: AAA: 6 (17%); Donor controls: Not known<br>Peripheral arterial disease: AAA: 6 (17%); Donor controls: Not known<br>Diabetes type 2: AAA: 3 (9%); Donor controls: Not known
Recruitment	Patients undergoing open elective surgery for AAA at the Karolinska University Hospital were consecutively recruited into the Stockholm AAA Biobank.Tissue samples were excised from the anterior vessel wall where intraluminal thrombus free locations. Patients with contagious diseases, reoperations, mycotic aneurysms, or genetic connective tissue diseases were not included. Control samples were taken from the abdominal aorta of beating-heart, brain-dead transplant donors.<br>There were no self-selection bias or other bias during the recruitment process.
Ethics oversight	Aortic samples from AAA patients were obtained from the surgeries performed at Karolinska Hospital in Stockholm. Signed consent from AAA patients was obtained for tissue collection. Control samples were taken from the abdominal aorta of beating-heart, solid organ transplant donors. Organ donors consented to the use of tissue for research purposes at the time of enlisting to the donation registry. Ethical permit was granted by Regional Ethical Review Board in Stockholm. No participant compensation was granted.

Note that full information on the approval of the study protocol must also be provided in the manuscript.

Field-specific reporting

Please select the one below that is the best fit for your research. If you are not sure, read the appropriate sections before making your selection.

☒ Life sciences ☐ Behavioural & social sciences ☐ Ecological, evolutionary & environmental sciences

For a reference copy of the document with all sections, see nature.com/documents/nr-reporting-summary-flat.pdf

Life sciences study design

All studies must disclose on these points even when the disclosure is negative.

Sample size	No statistical methods were used to pre-determine sample size. Sample size was approximated and derived from extensive publications within the cardiovascular field which were based on previous a priori power test.
Data exclusions	No data points were excluded.
Replication	Experiments were reproduced at least twice to confirm the obtained results.

Replication

There is one RNA-seq (flow on Ctrl vs AmotL2 KD HAoECs) performed on the samples coming from one experiment. According to the original plan, duplicates per group were enrolled and the experiments were performed twice independently. However, one of the experiment was discarded because the IBIDI pump was stopped for a few hours due to computer crush, even we re-connected and continued afterwards, KLF2 that serves as positive marker for flow responser, was not elevated after flow, Thus those samples were not sequenced.
After we obtained data from that one experiment (duplicates/group), we verified the key molecules of interest using qPCR on the samples collected from three other independent experiments (duplicates/group), in order to make sure the data is replicable.

Randomization

Animals were randomly assigned to experimental groups of different genotype.
For cell experiments, different dishes of cells were randomly assigned to experimental groups (scramble shRNA or Amotl2 shRNA; flow or static etc).

Blinding

For experiments in vitro, samples were harvested into the tubes with number labeling and then processed or analyzed to minimise the bias.
Mice were genotyped and assigned to different groups. For the mouse surgery and ultrasound, investigators were blind to mouse information.
Investigators who performed FACS sorting, scRNA-seq analysis, Proteomics, RNA-sequencing, and were blinded to the samples information.
For the experiments that was hard to blindly performed, for example IF image analyses, more than one investigators were invited to examine the images and do the quantification.

Reporting for specific materials, systems and methods

We require information from authors about some types of materials, experimental systems and methods used in many studies. Here, indicate whether each material, system or method listed is relevant to your study. If you are not sure if a list item applies to your research, read the appropriate section before selecting a response.

Materials & experimental systems

n/a	Involved in the study
☐	☒ Antibodies
☐	☒ Eukaryotic cell lines
☒	☐ Palaeontology and archaeology
☐	☒ Animals and other organisms
☒	☐ Clinical data
☒	☐ Dual use research of concern

Methods

n/a	Involved in the study
☒	☐ ChIP-seq
☐	☒ Flow cytometry
☒	☐ MRI-based neuroimaging

Antibodies

Antibodies used

For Immunostaining
GFP (Chk pAb to GFP) from Abcam (ab13970) 1:200
VE-cadherin (Purified anti human CD144, clone BV9) from Bio-legend (348502) 1:300
VE-cadherin (Purified rat anti mouse CD144, clone 11D4.1) from BD (555289) 1:300
CD31 (Pecam-1) (Purified rat anti mouse CD31, Clone MEC 13.3) from BD (553370) 1:300
CD45 (Mouse CD45 pAb) from R&D systems (AF-114) 1:300
p120-catenin (Purified Mouse Anti-p120 Catenin, Clone 98/pp120) from BD (610133) 1:100
Anti-Actin, α-Smooth Muscle (α-SMA) (Anti-Actin, α-Smooth Muscle - Cy3™ antibody, Mouse monoclonal, clone 1A4) from Sigma (C6198) 1:1000
TO-PRO-3 (TO-PRO- iodide (642/661)) from Life Technologies (T3605) 1:1000

For Western blot (WB)
β-actin (Ms mAb to Actin, clone mAbcam 8226) from Abcam (ab8226) 1:2000
VE-cadherin (Rabbit polyclonal VE Cadherin antibody) from Abcam (ab33168) 1:500
Lamin A/C (Monocolonal Anti-Lamin A/C ab produced in mouse, clone 4C11) from Sigma (SAB4200236) 1:500
Lamin B (Rb pAb to LaminB1) from Abcam (ab16048) 1:500
SUN2 (Rb mAb to SUN2, clone EPR6557) from Abcam (ab124916) 1:500
GAPDH (Monocolonal Anti-GAPDH ab produced in mouse, clone GAPDH-71.1) from Sigma (G8795) 1:3000
β-catenin (Purified mouse anti β-catenin, clone 14/Beta-Catenin) from BD (610154) 1:500
BirA (Anti-BirA antibody, clone 6C4c7) from Abcam (ab232732) 1:500

For both Immunostaining and Western blot (WB)
AmotL2 (Angiomotin like 2) from Innovagen, Lund, Sweden (catalog number: N/A). Purified from rabbit serum in Holmgren Lab. WB 1:500, IP 1:200, IF/IHC 1:100
p120-catenin (delta Catenin Monoclonal Antibody, clone 6H11) from Invitrogen (33-9700), WB 1:500 IF 1:100

For Fluorescence-activated cell sorting (FACS)
CD31 (FITC Rat Anti-Mouse CD31,Clone MEC 13.3) from BD (553372) 5ul/1000000 cells
CD45 (Alexa Fluor® 700 Rat Anti-Mouse CD45, Clone 30-F11) from BD (560510) 5ul/1000000 cells

For secondary antibodies used in Western blot (WB)
ECL® Anti-rabbit IgG HRP linked whole antibody from donkey, from GE Healthcare (NA934V) 1:10000
ECL® Anti-mouse IgG HRP linked whole antibody from donkey, from GE Healthcare (NA931V) 1:10000

ECL® Anti-rat IgG HRP linked whole antibody from donkey, from GE Healthcare (NA935V) 1:10000

For secondary antibodies used in Immunostaining
goat anti-Mouse IgG (H+L), Alexa Fluor® 405 conjugate from LifeTechnologies (A31553) 1:500
goat anti-Rat IgG (H+L), Alexa Fluor® 488 conjugate from LifeTechnologies (A11006) 1:500
donkey anti-Sheep IgG (H+L), Alexa Fluor® 488 conjugate from LifeTechnologies (A11015) 1:500
goat anti-Chicken IgG (H+L), Alexa Fluor® 488 conjugate from LifeTechnologies (A11039) 1:500
donkey anti-Goat IgG (H+L), Alexa Fluor® 488 conjugate from LifeTechnologies (A11055) 1:500
chicken anti-Rabbit IgG (H+L), Alexa Fluor® 488 conjugate from LifeTechnologies (A21441) 1:500
donkey anti-Mouse IgG (H+L), Alexa Fluor® 555 conjugate from LifeTechnologies (A31570) 1:500
donkey anti-Rabbit IgG (H+L), Alexa Fluor® 555 conjugate from LifeTechnologies (A31572) 1:500
goat anti-Mouse IgG (H+L), Alexa Fluor® 594 conjugate from LifeTechnologies (A11005) 1:500
goat anti-Rat IgG (H+L), Alexa Fluor® 594 conjugate from LifeTechnologies (A11007) 1:500
goat anti-Rabbit IgG (H+L), Alexa Fluor® 594 conjugate from LifeTechnologies (A11037) 1:500
goat anti-Rat IgG (H+L), Alexa Fluor® 633 conjugate from LifeTechnologies (A21094) 1:500
donkey anti-Mouse IgG (H+L), Alexa Fluor® 647 conjugate from LifeTechnologies (A31571) 1:500
donkey anti-Rabbit IgG (H+L), Alexa Fluor® 647 conjugate from LifeTechnologies (A31573) 1:500
goat anti-Mouse IgG (H+L), Cy3® from LifeTechnologies (A10521) 1:500
goat anti-Rabbit IgG (H+L), Cy3® from LifeTechnologies (A10520) 1:500

Validation

AmotL2 antibody was purified from rabbit serum using AmotL2 specific peptides by Innovagen company from Lund, Sweden. WB validation was performed in shControl and shAmotL2 treated HAoECs in Extended Data Fig.3a. IF validation was performed in aortic endothelial cells of amotl2 wild-type and knockout mice.
All the rest antibodies used in this study are commercialized and purchased from the company. The validation statements are available on the manufacturer's website.
GFP (Chk pAb to GFP) from Abcam (ab13970): https://www.abcam.com/products/primary-antibodies/gfp-antibody-ab13970.html
VE-cadherin (Purified anti human CD144, clone BV9) from Bio-legend (348502): https://www.biolegend.com/en-gb/products/purified-anti-human-cd144-ve-cadherin-antibody-6613
VE-cadherin (Purified rat anti mouse CD144, clone 11D4.1) from BD (555289): https://www.bdbiosciences.com/en-us/products/reagents/functional-cell-based-reagents/purified-rat-anti-mouse-cd144.555289
CD31 (Pecam-1) (Purified rat anti mouse CD31, Clone MEC 13.3) from BD (553370): https://www.bdbiosciences.com/en-us/products/reagents/flow-cytometry-reagents/research-reagents/single-color-antibodies-ruo/purified-rat-anti-mouse-cd31.553370
CD45 (Mouse CD45 pAb) from R&D systems (AF-114): https://www.rndsystems.com/products/mouse-cd45-antibody_af114
p120-catenin (Purified Mouse Anti-p120 Catenin, Clone 98/pp120) from BD (610133): https://www.bdbiosciences.com/en-us/products/reagents/microscopy-imaging-reagents/immunofluorescence-reagents/purified-mouse-anti-p120-catenin.610133
Anti-Actin, α-Smooth Muscle (α-SMA) (Anti-Actin, α-Smooth Muscle - Cy3™ antibody, Mouse monoclonal, clone 1A4) from Sigma (C6198): https://www.sigmaaldrich.com/SE/en/product/sigma/c6198
TO-PRO-3 (TO-PRO- iodide (642/661)) from Life Technologies (T3605): https://www.thermofisher.com/order/catalog/product/T3605
β-actin (Ms mAb to Actin, clone mAbcam 8226) from Abcam (ab8226): https://www.abcam.com/products/primary-antibodies/beta-actin-antibody-mabcam-8226-loading-control-ab8226.html
VE-cadherin (Rabbit polyclonal VE Cadherin antibody) from Abcam (ab33168): https://www.abcam.com/products/primary-antibodies/ve-cadherin-antibody-intercellular-junction-marker-ab33168.html
Lamin A/C (Monocolonal Anti-Lamin A/C ab produced in mouse, clone 4C11) from Sigma (SAB4200236): https://www.sigmaaldrich.com/SE/en/product/sigma/sab4200236
Lamin B (Rb pAb to LaminB1) from Abcam (ab16048): https://www.abcam.com/products/primary-antibodies/lamin-b1-antibody-nuclear-envelope-marker-ab16048.html
SUN2 (Rb mAb to SUN2, clone EPR6557) from Abcam (ab124916): https://www.abcam.com/products/primary-antibodies/sun2-antibody-epr6557-ab124916.html
GAPDH (Monocolonal Anti-GAPDH ab produced in mouse, clone GAPDH-71.1) from Sigma (G8795): https://www.sigmaaldrich.com/SE/en/product/sigma/g8795
β-catenin (Purified mouse anti β-catenin, clone 14/Beta-Catenin) from BD (610154): https://www.bdbiosciences.com/en-us/products/reagents/microscopy-imaging-reagents/immunofluorescence-reagents/purified-mouse-anti-catenin.610154
BirA (Anti-BirA antibody, clone 6C4c7) from Abcam (ab232732): https://www.abcam.com/products/primary-antibodies/bira-antibody-6c4c7-ab232732.html
p120-catenin (delta Catenin Monoclonal Antibody, clone 6H11) from Invitrogen (33-9700): https://www.thermofisher.com/antibody/product/delta-Catenin-Antibody-clone-6H11-Monoclonal/33-9700
CD31 (FITC Rat Anti-Mouse CD31,Clone MEC 13.3) from BD (553372): https://www.bdbiosciences.com/en-us/products/reagents/flow-cytometry-reagents/research-reagents/single-color-antibodies-ruo/fitc-rat-anti-mouse-cd31.553372
CD45 (Alexa Fluor® 700 Rat Anti-Mouse CD45, Clone 30-F11) from BD (560510): https://www.bdbiosciences.com/en-us/products/reagents/flow-cytometry-reagents/research-reagents/single-color-antibodies-ruo/alexa-fluor-700-rat-anti-mouse-cd45.560510

Eukaryotic cell lines

Policy information about cell lines and Sex and Gender in Research

Cell line source(s)

Murine endothelial cells, MILE SVEN 1 (MS1), were purchased from ATCC (CRL-2297). MS1 is a pancreatic islet endothelial cell line established in 1994 from female C57BL/6 strain mice.
Bovine aortic endothelial (BAE) cells were purchased from Sigma-Aldrich (B304-05). BAE cells are primary cells from aorta of female bovine.
Human Aortic Endothelial Cell (HAoECs) were purchased from PromoCell (C-12271). The batch of HAoECs used for this study came from a 55-year-old male donor with a Caucasian background.
Human Umbilical Vein Endothelial Cells (HUVECs) were purchased from ScienCell (#8000). The company doesn't provide the sex information.

Authentication	All those cell lines or primary cells were purchased from the company. They were not authenticated afterwards.
Mycoplasma contamination	The cells were not tested for mycoplasma contamination. There was no signs of DNA contamination while visualizing DAPI in IF staining.
Commonly misidentified lines (See ICLAC register)	There is no commonly misidentified cell lines were used in the study.

Animals and other research organisms

Policy information about studies involving animals; ARRIVE guidelines recommended for reporting animal research, and Sex and Gender in Research

Laboratory animals	All the mice in this report had C57BL/6 background. The strain is amotl2flox/flox / Cdh5(PAC)CreERT2 / ROSA26-EYFP.<br>amotl2flox/flox mice were purchased from TaconicArtemis GmbH.<br>Cdh5(PAC)CreERT2 / ROSA26-EYFP was kindly provided by Dr. Ralf H Adams, University of Münster.<br>Mice were housed in standard vented cages in rooms with controlled temperature (20-22°C) and humidity (40-60%) with 12-hour light-dark cycles, and free access to water and food.<br><br>Summary of mouse age :<br>7-9 months old mice were selected for AmotL2 expression in WT aorta analysis (Fig 1c and Extended Data Fig 1a), descending aorta/ vena cava/urine bladder (WT/KO) EC morphology analyses (Fig. 2a-l), co-immunoprecipitation of VE-cadherin in WT mice lung (Extended Data Fig. 4b), descending aorta (WT/KO) RNA-seq and qPCR analysis (Fig. 5b-f and Extended Data Fig. 6a-e), Cd45 cells infiltration in mouse aortae (Fig. 5g and Extended Data Fig. 6f-i), murine aortic aneurysm (WT/KO) analysis (Fig. 7a-b) and ascending/ descending aorta RNA-seq/Proteomic analysis in WT mice (Extended Data Fig. 9a-f).<br><br>12-week-old mice were used for AmotL2 retina staining (Extended Data Fig. 1d,f), PLA staining in DA/vena cava (Fig. 4d,e), Single-cell RNA-seq analysis (Fig. 6a-e and Extended Data Fig. 7a-g) and PPE (Porcine Pancreatic Elastase) aortic aneurysm model (Fig. 7c-g and Extended Data Fig. 9g-i).<br><br>Postnatal day 6 mice were used for AmotL2 retina staining (Extended Data Fig. 1d,e)
Wild animals	There were no wild animals used in this study.
Reporting on sex	Both females and males were included.
Field-collected samples	There were no field-collected samples used in this study.
Ethics oversight	Ethical permits were approved by Stockholm North Animal Experiment Ethics Board and all experiments were carried out in accordance with the guidelines of the Swedish Board of Agriculture.

Note that full information on the approval of the study protocol must also be provided in the manuscript.

Flow Cytometry

Plots

Confirm that:

☒ The axis labels state the marker and fluorochrome used (e.g. CD4-FITC).

☒ The axis scales are clearly visible. Include numbers along axes only for bottom left plot of group (a 'group' is an analysis of identical markers).

☒ All plots are contour plots with outliers or pseudocolor plots.

☒ A numerical value for number of cells or percentage (with statistics) is provided.

Methodology

Sample preparation	The mice were euthanized and the aorta was dissected out after PBS perfusion. Opened aortae were placed in digesting buffer at 37 degree for 30 minutes. Cell mixtures were negatively selected by Cd45 magnetic beads after 15 minute room temperature incubation. Single cell suspension was stained, on ice, for 30 min with Cd31 and Cd45 antibodies. Stained cells were washed with 1X PBS and died with viability dye. Incubated for 20 min before FACS.
Instrument	Sony SH800S
Software	Sony Cell Sorter Software, Version 2.1.3
Cell population abundance	In Cd45 depleted cell suspensions.<br>AmotL2ec-/ec- cell abundance: Cd31+ Cd45- cells were 0.91% , Cd31- Cd45- 8.39% and Cd45+ 0.7%.<br>AmotL2ec+/ec+ cell abundance: Cd31+ Cd45- cells were 0.70%, Cd31-Cd45- 99.22% and Cd45+ 0.08%.<br>Cell types were verified with expression of endothelial markers (CD31 and Cdh5) after single cell sequencing had been performed on sorted populations.

Gating strategy	FSC-A vs BSC-A was used to separate cells from debris, and FSC-H vs FSC-W to ensure that only singlets were sorted. Then eFluor-450 vs FSC-A was used to find eFluor450 negative cell population (viable), which is lower than 10^5 on the log scale. Cd45 vs Cd31 was plotted and cells with values lower than 10^2.5 (on the bioexponential scale) were determined negative in regards to Cd45 and cells with lower than 10^4 (on the bioexponential scale) were determined as CD31 negative.

☒ Tick this box to confirm that a figure exemplifying the gating strategy is provided in the Supplementary Information.
