# Supplementary figures and images for: The VE-cadherin/AmotL2 mechanosensory pathway suppresses aortic inflammation and the formation of abdominal aortic aneurysms
Source: Nat Cardiovasc Res. 2023 Jun 29;2(7):629–44. doi: 10.1038/s44161-023-00298-8 (PMC11358041; doi:10.1038/s44161-023-00298-8)

Full length blots to Fig. 4b.

HUVEC

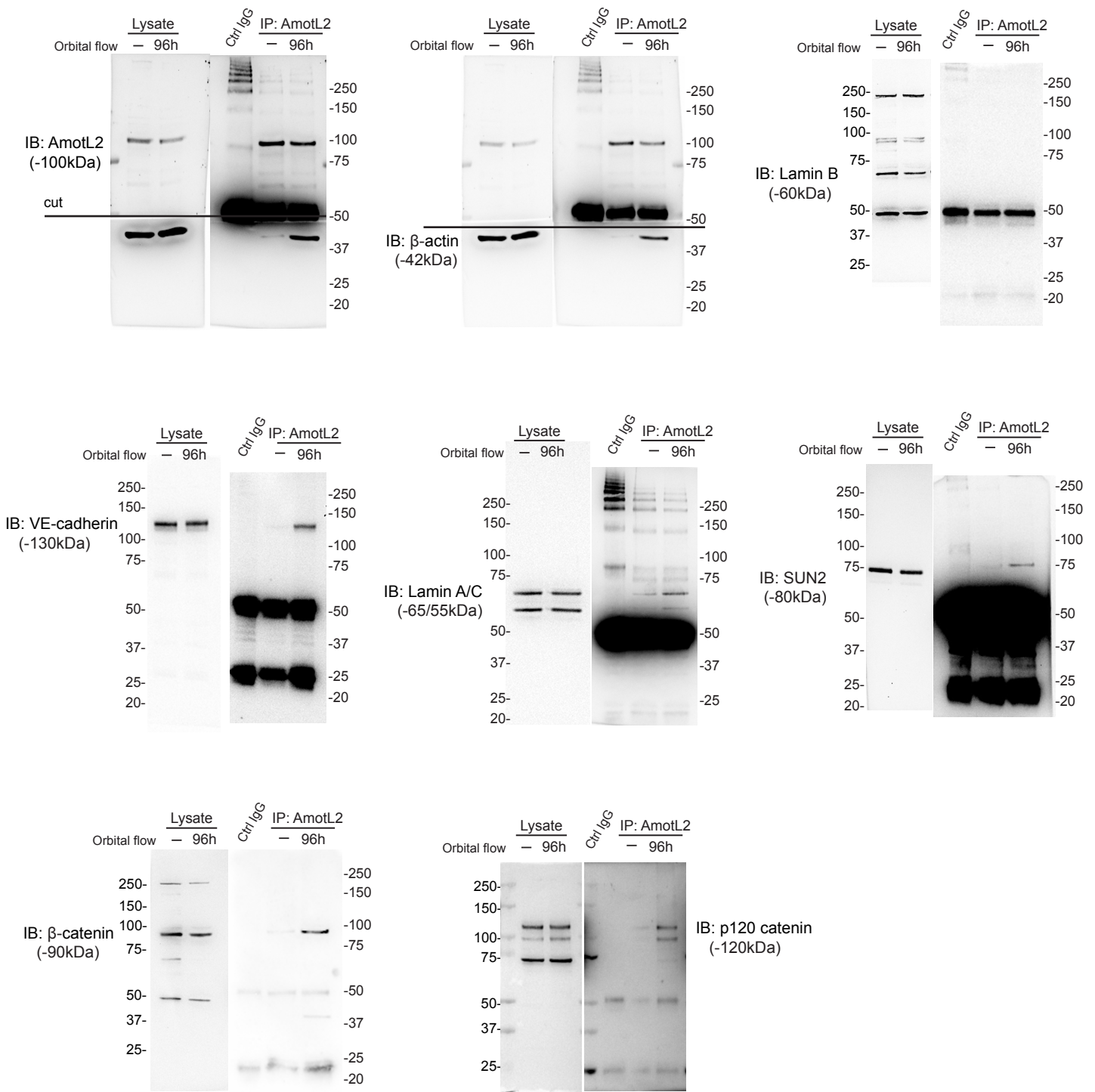

Full length blots to Fig. 4b.

HAoEC

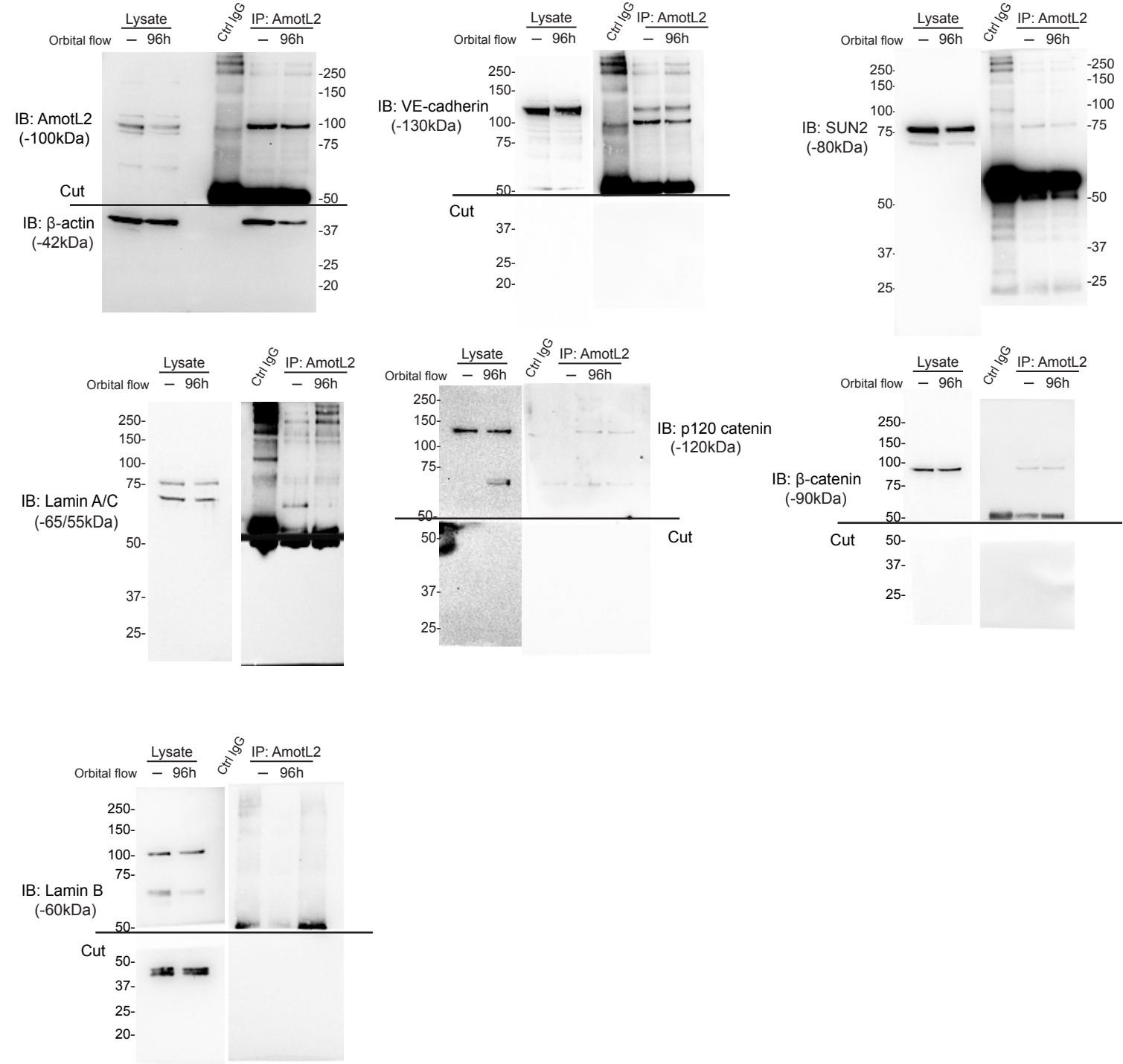

Supplement: Supplementary file 17 — Unprocessed western blots [file 44161_2023_298_MOESM17_ESM.pdf]

**Extended Data Fig. 3a**

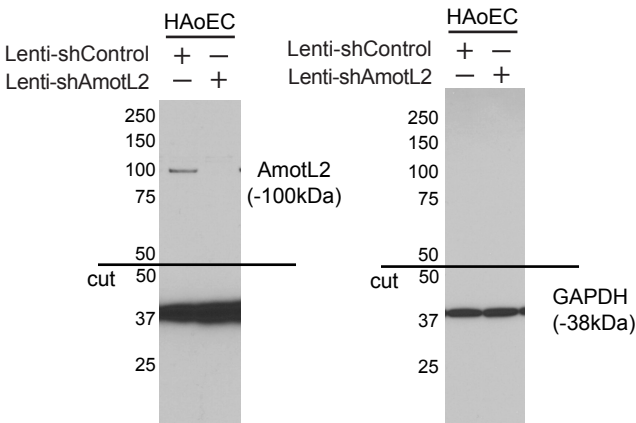

**Extended Data Fig. 3f**

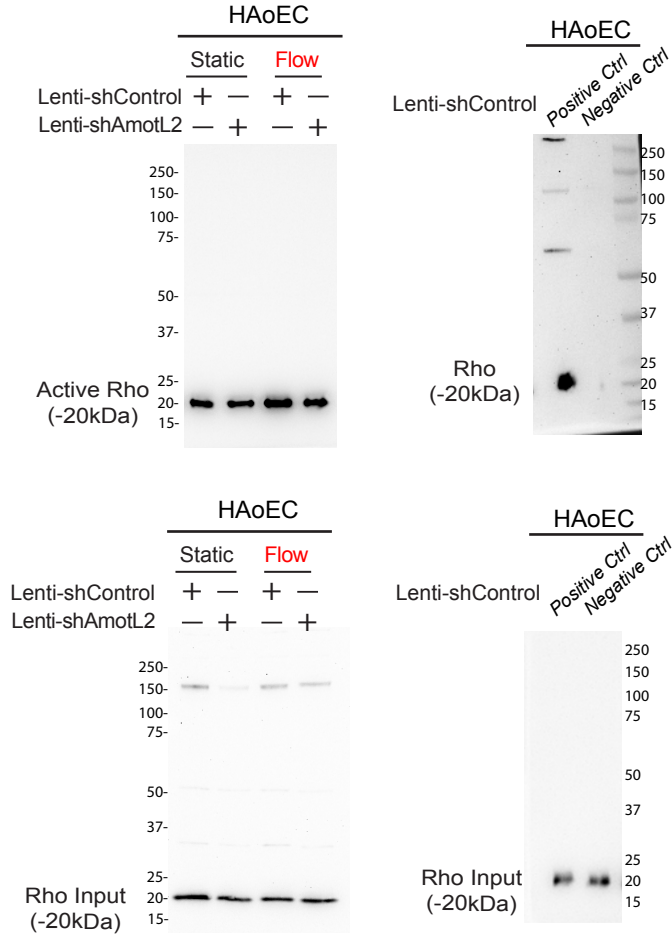

Supplement: Supplementary file 25 — Unprocessed western blots [file 44161_2023_298_MOESM25_ESM.pdf]

Full length blots to **Extended Data Fig. 4b**

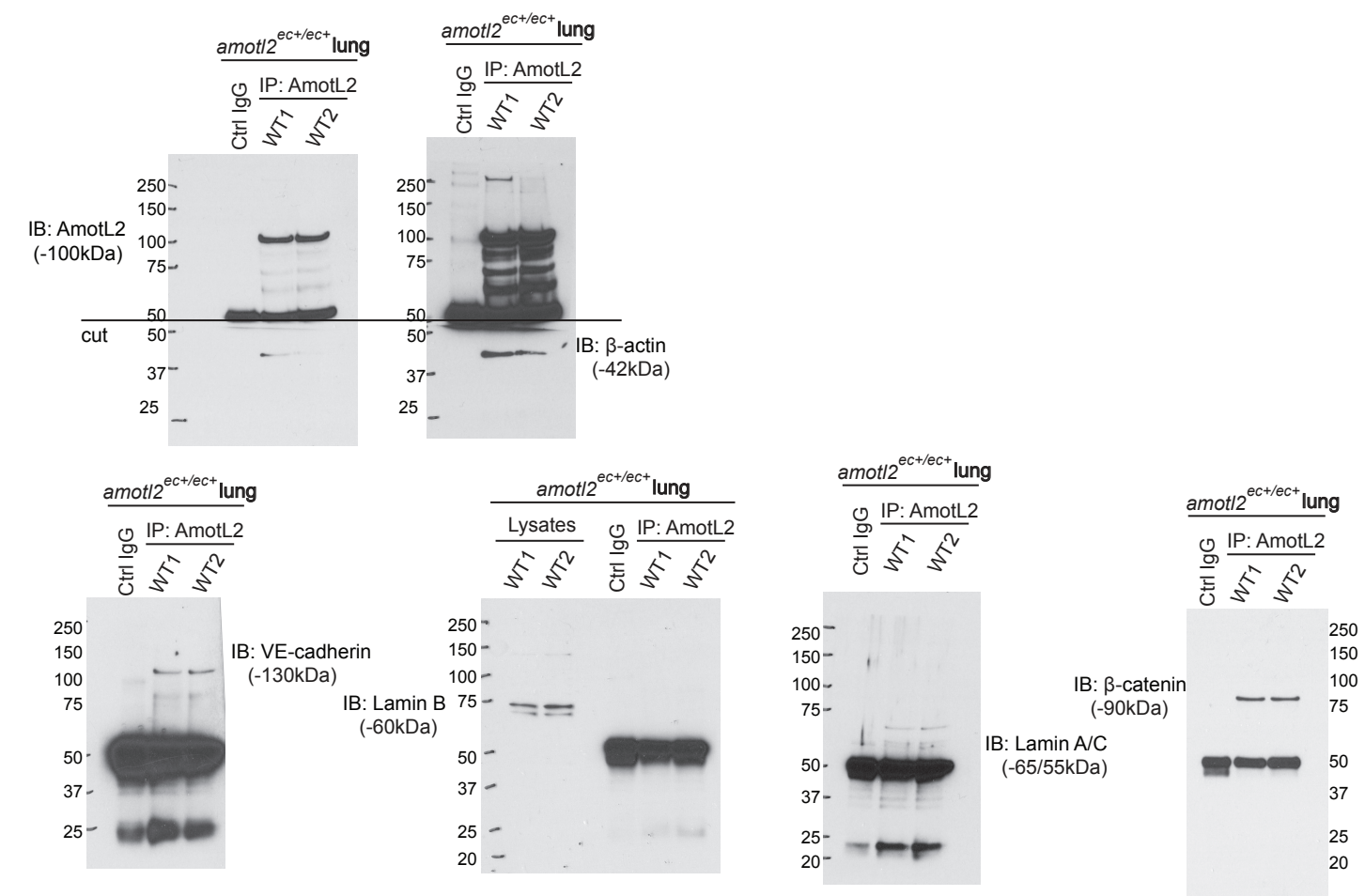

Supplement: Supplementary file 27 — Unprocessed western blots [file 44161_2023_298_MOESM27_ESM.pdf]

Full length blots to **Extended Data Fig. 5b** and **5d**.

**Extended Data Fig. 5b**

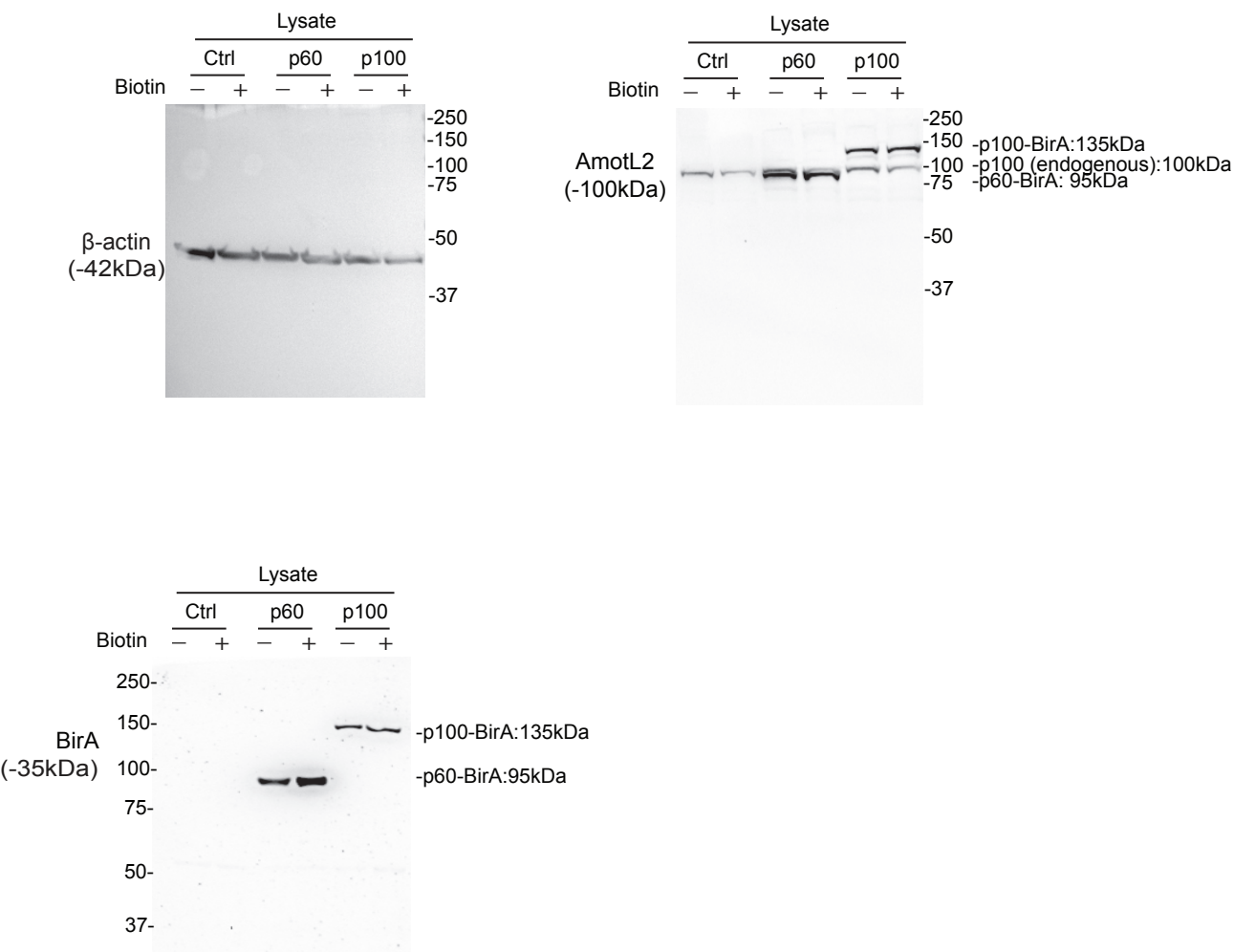

**Extended Data Fig. 5d**

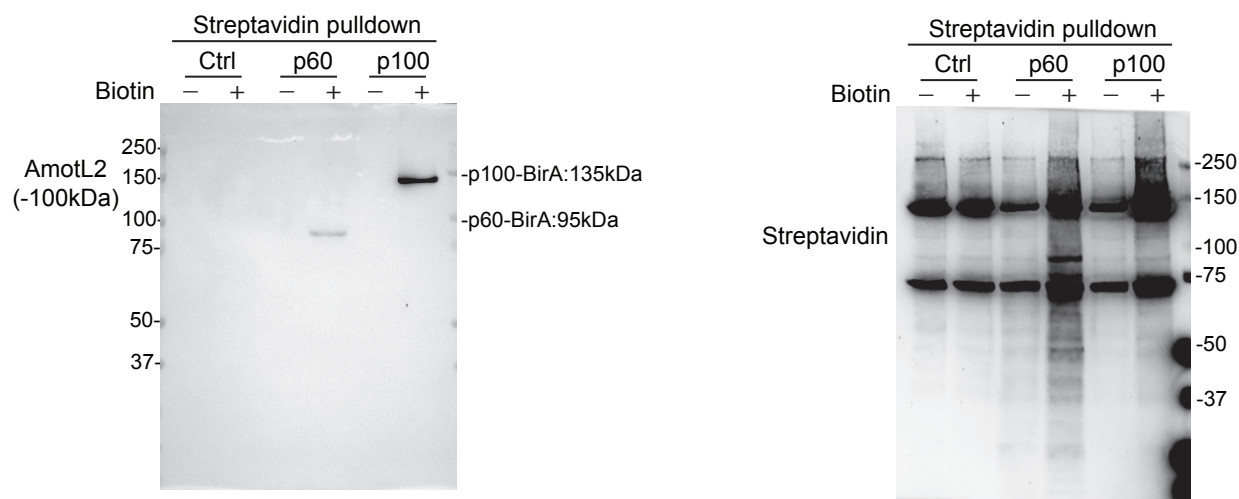

Supplement: Supplementary file 28 — Unprocessed western blots [file 44161_2023_298_MOESM28_ESM.pdf]
